# Towards sub-nanometer real-space observation of spin and orbital magnetism at the Fe/MgO interface

**DOI:** 10.1038/srep44802

**Published:** 2017-03-24

**Authors:** Thomas Thersleff, Shunsuke Muto, Mirosław Werwiński, Jakob Spiegelberg, Yaroslav Kvashnin, Björgvin Hjӧrvarsson, Olle Eriksson, Ján Rusz, Klaus Leifer

**Affiliations:** 1 Department of Engineering Sciences, Uppsala University, Box 534, 75121 Uppsala, Sweden; 2 Advanced Measurement Technology Center, Institute of Materials and Systems for Sustainability, Nagoya University, Chikusa-ku, Nagoya 464-8603, Japan; 3 Department of Physics and Astronomy, Uppsala University, Box 516, 75120 Uppsala, Sweden; 4 Institute of Molecular Physics Polish Academy of Sciences, M. Smoluchowskiego 17, 60-179 Poznań, Poland

## Abstract

While the performance of magnetic tunnel junctions based on metal/oxide interfaces is determined by hybridization, charge transfer, and magnetic properties at the interface, there are currently only limited experimental techniques with sufficient spatial resolution to directly observe these effects simultaneously in real-space. In this letter, we demonstrate an experimental method based on Electron Magnetic Circular Dichroism (EMCD) that will allow researchers to simultaneously map magnetic transitions and valency in real-space over interfacial cross-sections with sub-nanometer spatial resolution. We apply this method to an Fe/MgO bilayer system, observing a significant enhancement in the orbital to spin moment ratio that is strongly localized to the interfacial region. Through the use of first-principles calculations, multivariate statistical analysis, and Electron Energy-Loss Spectroscopy (EELS), we explore the extent to which this enhancement can be attributed to emergent magnetism due to structural confinement at the interface. We conclude that this method has the potential to directly visualize spin and orbital moments at buried interfaces in magnetic systems with unprecedented spatial resolution.

As the spatial dimensions of a magnetic material are reduced below the characteristic lengths of individual magnetic domains and the structural symmetry of a magnetic material is broken at an interface, it becomes possible to achieve novel magnetic behavior that may be unobtainable in bulk systems. These properties can be upscaled to macroscopic materials through the fabrication of magnetic heterostructures and used to control the spin structure of electrical transport devices. Understanding this behavior requires experimental techniques that quantitatively describe magnetic moments while simultaneously pin-pointing interesting variations to nanoscale features in real-space. While a quantitative description of magnetic moments can be achieved with x-rays through the use of X-Ray Magnetic Circular Dichroism (XMCD)^1^, difficulties inherent in focusing soft x-rays down to the sub-10 nm regime^2^ leave these length scales relatively unexplored experimentally. Consequently, interfaces can only be investigated through careful sample fabrication and diffraction experiments^3^, and these necessarily probe surface areas on the order of microns to millimeters, requiring assumptions to be made about the macroscale homogeneity of the investigated samples.

One example of a system where knowledge of interfacial magnetism plays a critical role in the development of applications is the metal/oxide Fe/MgO interface used in Magnetic Tunnel Junctions (MTJs). While the Tunnel Magnetoresistance (TMR) in this system is predicted to exceed 1000%^4^, local structural and chemical fluctuations on the nanoscale are believed to limit experimental values to around 200%^5^,^6^. Thanks in part to the use of electron radiation techniques, the local structural^7^,^8^,^9^, electronic, and chemical^10^,^11^ properties have been extensively investigated with up to atomic resolution. However, these properties have only been indirectly linked to emergent magnetic effects^3^. An improved understanding of this system requires the development of an experimental technique that can directly link magnetism to nanoscale structural and chemical variations.

In this letter, we use an angular-resolved Electron Energy-Loss Spectroscopy (EELS) technique known as Electron Magnetic Circular Dichroism (EMCD)^12^ to experimentally probe spin and orbital magnetism at the Fe/MgO interface in real-space with a spatial resolution of approximately 0.8 nm. We observe an experimental increase in the orbital *m*_*L*_ to effective spin 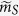 magnetic moment ratio 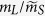 in proximity to the interface. First principles simulations based on Density Functional Theory (DFT)^13^ combined with Dynamical Mean-Field Theory (DMFT)^14^,^15^ show that such behavior could be partially explained by a reduction in crystalline symmetry caused by an atomically sharp interfacial bond between iron and oxygen atoms. While this interfacial model is supported experimentally by High Angle Annular Dark Field (HAADF) Scanning Transmission Electron Microscopy (STEM) and Electron Energy Loss Spectroscopy (EELS) investigations, we note that the observed increase is larger than predicted by theory. This leads us to explore whether non-magnetic phenomena could influence the observed 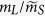 enhancement, concluding that magnetism is likely to constitute a dominant contribution, but may be entangled with charge transfer effects due to interfacial bonding between iron and oxygen atoms. We believe that further refinements to this experimental design could distinguish these effects, allowing researchers to directly explore the local magnetic properties of magnetic interfaces, opening the way for considerably more detailed investigations into the influence of emergent interfacial effects on spintronic materials in the future.

## Results

**Figure 1:**
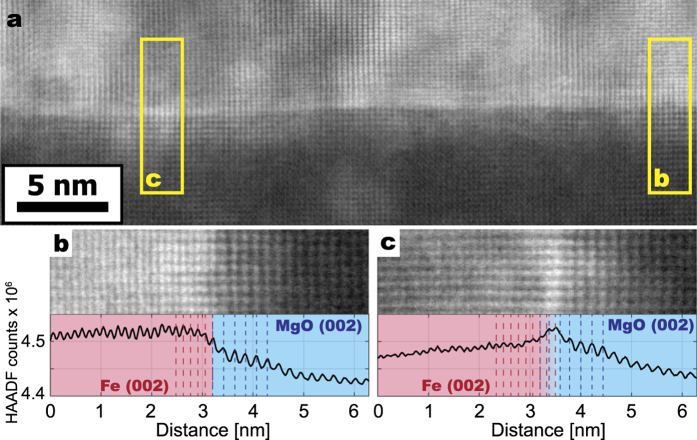
(**a**) A high resolution HAADF-STEM micrograph of the Fe/MgO interface. The contrast mechanism is predominately Z-contrast with MgO at the bottom and Fe at the top. Yellow boxes denote the regions from which line scans are extracted. In (**b**), the line scan shows a sharp interface with minimal overlap of the Fe (0 0 2) and MgO (0 0 2) lattice planes. In (**c**), a more pronounced overlap is observed, likely due to a projection effect of the stepped interface.

A 50 nm thin film of bcc iron was epitaxially grown on top of a single crystal MgO (0 0 1) substrate using Molecular Beam Epitaxy (MBE). The structure of the interface was investigated using high resolution HAADF-STEM and is summarized in Fig. 1. In panel a, the full STEM image is presented. The pixel intensity scales approximately as 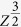 where *Z* is the atomic number. At the interface with Fe, a faint horizontal streaking effect parallel to the substrate surface is observed with a periodicity of about 1 nm. This is a Moiré contrast and it has been observed at this interface previously^7^. Line scans from two regions denoted in yellow boxes are presented in panels b and c of Fig. 1. In panel b, the line scan reveals two distinct regions of Fe (0 0 2) lattice planes that interface sharply with MgO (0 0 2) planes, suggesting that there are no secondary phases present. In panel c, these two lattice planes overlap to some extent, suggesting that the interface is not perfectly parallel to the direction of beam propagation. We interpret this as arising from atomic steps in the surface of the MgO substrate, which are known to form due to the fabrication conditions utilized for this sample^7^. Subsequent high resolution investigations of this interface are presented in the supplementary information and show that the step size of the MgO surface can reach up to 2 nm in height. From this, we infer that the interface is best described as free of secondary phases yet projected as a series of steps along the direction of beam propagation in the lamella, consistent with the findings of previous investigators^3^,^7^,^8^.

**Figure 2:**
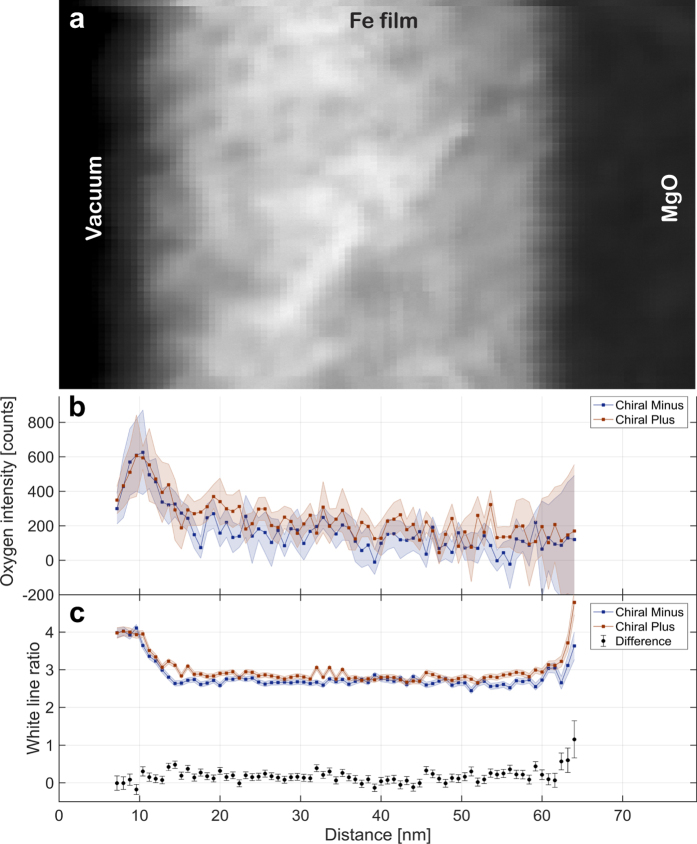
Chemical analysis of the region investigated with EMCD. In (**a**), the HAADF survey image is provided as a reference for the line profiles below. Following the vertical summation of all individual spectra, the integrated intensity of the oxygen pre-peak for the oxide cladding layers is shown in (**b**) for both chiral positions while the white line ratio for the iron edges for both chiral positions is presented in (**c**).

The chemistry of the interface was investigated using EELS and is summarized in Fig. 2. In Fig. 2a, the HAADF survey image is provided and the vacuum, Fe film, and MgO regions are labeled. Figure 2b presents the integrated intensity under the oxygen pre-peak energy range (526–533 eV) for the two aperture positions needed for EMCD analysis after vertically summing all of the individual spectra into a line scan. The oxygen pre-peak is known to be closely related to the presence of iron oxide^16^, allowing for a separation of the iron oxide species from the oxygen in the MgO substrate. A strong increase in the oxygen pre-peak signal for both aperture positions is visible at the vacuum/Fe interface while no statistically significant increase is observed at the Fe/MgO interface. Moreover, there appears to be no statistically significant increase in the overall oxygen concentration between the two independent scans, indicating that *in-situ* oxidation between scans is likely minimal. This analysis is detailed further in the supplementary information.

Figure 2c shows the ratio of the integrated intensities under the Fe *L*_3_ and Fe *L*_2_ edges^7^,^17^, also known as the white line ratio. This ratio clearly increases at the interface for both aperture positions within the margin of error. The difference in white line ratio for both aperture positions is plotted in the lower part of this graph. Throughout the bulk of the sample, the difference is greater than zero, which can be interpreted to arise from chiral scattering effects that give rise to EMCD. Moreover, a strong increase in this difference is observed at the interfacial region. Such behavior has been previously noted at the edge of a nanoparticle and was interpreted as qualitative evidence in favor of changes in spin orbital ratios^18^. We interpret these data in the same manner while noting that this method alone cannot be used to quantitatively describe the presence of a magnetic circular dichroic effect.

To investigate interfacial magnetism, we have apply the EMCD technique^12^ in such a way to generate real-space maps of magnetic transitions with sub-nanometer spatial resolution. EMCD exploits the fact that the inelastic scattering of electrons is influenced by the presence of uncompensated magnetic moments. This manifests itself as an antisymmetric signal component in the diffraction plane, originating from dipole transitions in a manner analogous to X-Ray Magnetic Circular Dichroism (XMCD). Sum rules can be applied to the resultant difference spectra, known as the “EMCD signal”, yielding quantitative information about magnetic transitions, such as the ratio of orbital to effective spin magnetic moments 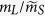
19,20. With electrons as a radiation source, the probed volumes can be on the order of tens of cubic nanometers^21^. Additionally, existing STEM infrastructure can be exploited to deploy the technique in scanning microdiffraciton mode^18^,^21^,^22^,^23^. This allows for the acquisition of multiple complimentary hyperspectral datacubes from the same sample region but with different electron scattering momenta, in a technique we call STEM-EMCD.

**Figure 3:**
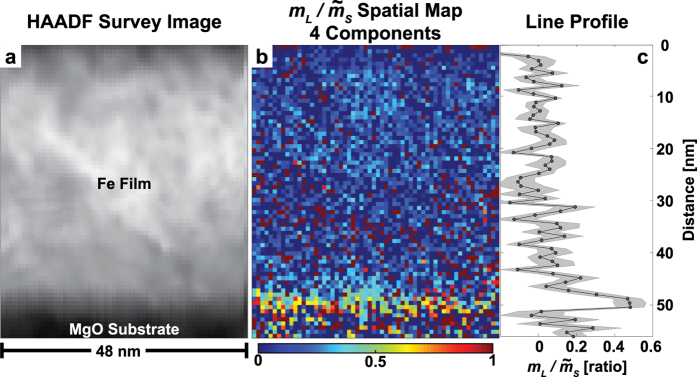
Real-space map of magnetic moments in the iron thin film. The HAADF survey image in (**a**) shows a primarily Z-contrast representation of the iron film and MgO substrate. In (**b**), the 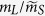 real-space map is presented on a color scale from 0 to 1. A line profile of this map is shown in (**c**) with error bars representing ±1 standard deviation of values for each row.

Two individual datacubes with conjugated electron scattering momenta (here referred to as “chiral plus” and “chiral minus”) were acquired from the region shown in the HAADF micrograph presented in Fig. 3. While similar chiral datasets have previously been used to qualitatively infer spatially-resolved magnetic behavior in nanoparticles^18^ as well as quantitatively extract 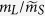 with high precision from the entire scanned region^22^, the low signal to noise ratio in individual spectra has precluded the completion of both goals simultaneously. To accomplish this, we exploit spectral redundancy of the datacube to approximate the raw data by using the principal spectral components of highest variance. In this case, the robust principal component analysis (ROBPCA)^24^ algorithm was applied to the two chiral datacubes individually. These were then treated as detailed in the methods section and the sum rules applied on a pixel-by-pixel basis, resulting in a map of 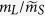 with the same pixel resolution as the original dataset. The results for a four component reconstruction are presented in Fig. 3. Scree plots, loading curves, score maps, and real-space EMCD reconstructions for the first ten components are provided in the supplementary information. In each of these maps, the pixel size is approximately 0.8 nm, with minimal beam broadening expected due to the low convergence angle, as detailed in the supplementary information. Within a 2.4 nm wide region near the Fe/MgO interface, a clear enhancement of 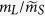 is observed, and this is consistent for all map reconstructions up to six components (see supplementary). This closely corroborates the results of Fig. 2 and suggests that 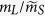 at the interface may be higher than the bulk value.

**Figure 4:**
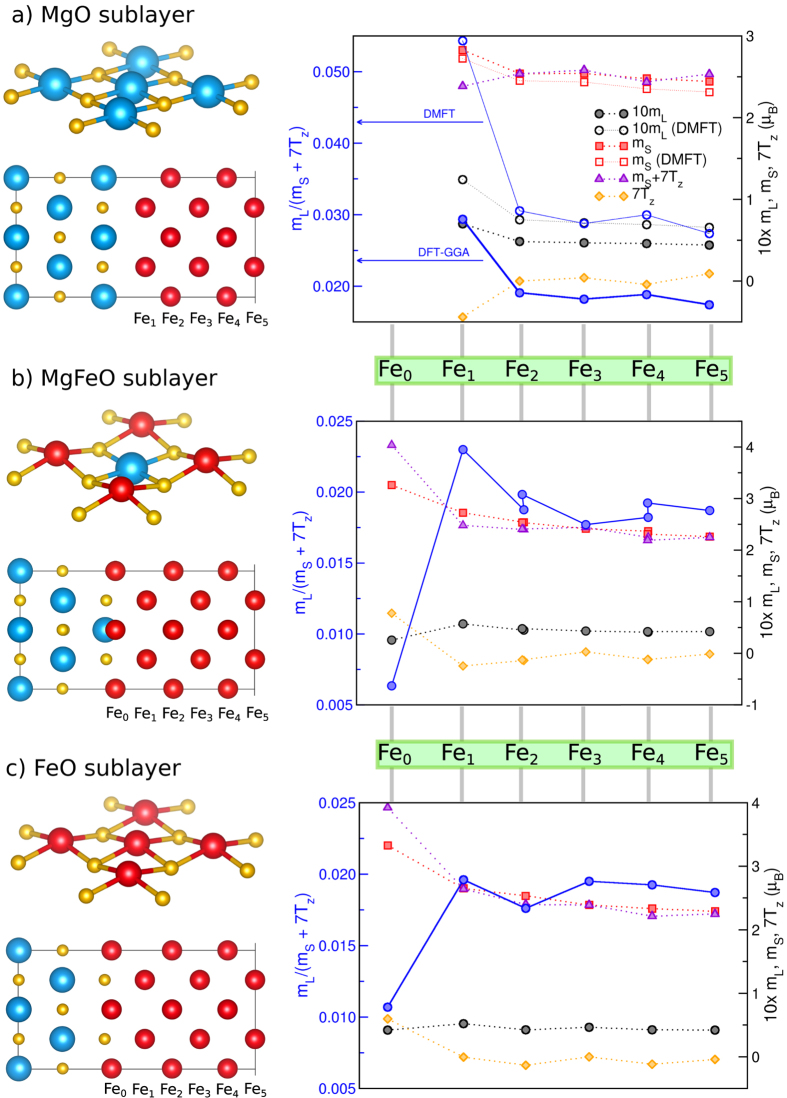
Three structure models of an MgO/Fe interface with various level of intermixing. Model (**a**) considers an atomically sharp interface, while models (**b**) and (**c**) consider 50% and 100% intermixing of Fe with Mg atoms in the topmost MgO layer, respectively. The right panels summarize calculated magnetic properties of individual Fe layers: their spin moments *m*_*s*_, orbital moments *m*_*L*_, magnetic dipole terms *T*_*Z*_ (right vertical axis), and resulting effective orbital to spin moment ratio 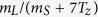 (left vertical axis, denoted by filled (DFT-GGA) or open (DMFT) blue circles).

To explore whether the origin of this perceived increase in 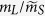 could be due to interfacial magnetism effects, we employed theoretical simulations. Three interface models tractable with electronic structure calculations were considered: one atomically sharp interface, where the iron atoms are bonded to oxygen atoms of the top-most layer of the MgO substrate^11^, and two models where an intermixing of the iron into the MgO surface layer is considered. The intermixing models consist of a Mg_0.5_Fe_0.5_O layer, where 50% of the Mg atoms are replaced by Fe, and an FeO layer, where all of the Mg atoms are replaced by Fe. We consider these models to represent the boundary cases for a sharp interface with none versus complete intermixing of Mg and Fe atoms. Structural optimization was performed within DFT for all model interfaces and the local magnetic properties were extracted. The results are summarized in Fig. 4. The DFT calculations reveal an interesting dependence of the magnetic moments on the degree of Fe-MgO intermixing. If the interface is atomically sharp and no intermixing occurs, then both the spin *m*_*s*_ and orbital *m*_*L*_ moments of Fe atoms near the interface grow. The enhancement of the orbital moment is larger, leading to an increase of the *m*_*L*_/*m*_*s*_ ratio at the interface. The value of the interfacial Fe moment obtained for this structure model is between 2.84 and 2.9 *μ*_*B*_ depending on the calculation, which is in reasonable agreement with XMCD-derived estimate of 3.3 ± 0.3 *μ*_*B*_^25^. A prior theoretical study^26^ reports a slightly larger *m*_*L*_ value at the interface from plain DFT calculation, which we attribute to the differences in the used electronic structure codes. Moreover, according to the sum rules^27^, the experimentally accessible quantity is the orbital to *effective* spin moment ratio 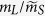, where 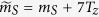. The latter contribution denotes the expectation value of the spin dipole moment operator (*T*_*z*_), which is negligible in cubic materials^27^, but can be appreciable in e.g. oxides^28^. The results shown in Fig. 4 clearly indicate that the reduction of the local symmetry at the interface causes the magnetic dipole term 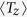 to grow significantly. In the case of the atomically sharp interface, it takes on a negative sign, which further reduces the denominator in the sum rules expression leading to a further enhancement of the observed 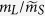 ratio from approximately 0.03 to 0.05 at the interface. This finding is also confirmed in detail by DFT+DMFT calculations, which employ a more sophisticated treatment of the correlation effects on Fe sites and results in improved values of orbital moments as compared to bare DFT^29^ (see Fig. 4 as well as the supplementary information). A relatively large negative value of 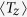 obtained for the first structure model is consistent with previous results for Fe surface^30^ under the assumption that Fe interacts weekly with MgO, as was suggested by Li *et al*.^31^. In contrast, both the intermixing interfacial models suggest that the spin moment grows and the orbital moment shows first an increase near the interface followed by a decrease within the mixed layer. Moreover, the 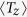 term remains positive, meaning that the observed effective 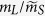 ratio is likely to decrease.

## Discussion

While the DFT and DMFT simulations support the experimental observation of an enhanced 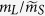 at the interface, there are some important discrepancies that need to be discussed in detail before conclusions are drawn. First, we note that the spatial extension of the interfacial region with increased white line ratio change is (2–3 nm, and this is larger than the single monolayer predicted by DFT and DMFT simulations. However, the white line and branching ratio of this interface is also expected to increase over a monolayer of material, yet is experimentally observed to be extended by approximately this amount as well^3^,^7^,^8^. We can understand these observations as a consequence of a three-dimensional projection effect caused by the finite thickness of the TEM lamella and the interfacial roughness of the Fe/MgO interface. In HRTEM images (see supplementary information), we observe an interfacial roughness of the Fe/MgO interface in the range of a few nanometers, consistent with previous reports^7^. Hence, we conclude that the EELS signal from interfacially bonded iron is convolved with the EELS signal from bulk iron over a range of some nanometers, and that this effect seems to be characteristic to this system. The detailed description of the relationship between interfacial width and the detected EMCD signal is discussed in greater detail in the supplementary information.

Second, the simulated enhancement of 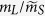 at the interface is approximately twice of the bulk value, whereas experiments put this at closer to four times. Here, we note that the large spread of 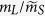 values complicates the direct utilization of the absolute value with high confidence. Nevertheless, a qualitative increase in mean 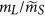 at the interface appears to be statistically significant (see the supplementary information for more details). While most systematic errors that could give rise to this locally-observed relative change can be ruled out by serial nature of the experimental design and the collection of large numbers of independent spectra, there are some experimental limitations that need more consideration. These limitations are a consequence of the fact that a minimum of two independent datacubes are needed to obtain an EMCD signal, and that these are compared on a pixel-by-pixel basis. For example, if the exact same region is not being sampled between the chiral plus and chiral minus datacubes, it is possible that one scan would have a higher percentage of interfacial atoms than the other. This could result in a white line ratio modification that is not related to magnetism but is very challenging to detect, leading to the perception of an increase in 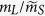, particularly at the interface. However, this alone cannot account for our observed increase in 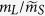. We do not observe large spatial shifts in these datacubes and have even purposefully shifted the two datacubes with respect to one another to understand this effect better (see the supplementary information). Our assessment is that, since the 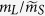 enhancement takes place over a relatively large distance, such spatial drift has a small impact on the relative values and is not of major concern. Regardless, we cannot rule out sub-pixel drifts or more complex drift patterns beyond pure translation, and this may result in some error for the estimation of interfacial 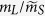. Another experimental challenge is the possibility that the sample dynamically oxidizes between scans due to the decomposition of the MgO substrate. We anticipated this problem in the design of our experiment, limiting the electron dose while maximizing the collection efficiency. Figure 2 indicates that *in situ* oxidation effects were minimal, and HAADF survey images taken prior to and after the experiment (see supplementary information) do not reveal any strong beam damage effects before or after our experiments. More details are provided in the supplementary information. Despite this, we are cautious to conclude that these error sources can be completely ruled out, and therefore consider them to be a plausible explanation for at least some of the observed enhancement in 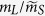. Here we note that future experiments with improved spatial registration or investigations on systems that are not subject to strong oxidation effects could allow for a better separation between charge transfer effects and magnetism.

Finally, we note that the absolute values for 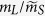 appear to depend somewhat on the experimental acquisition geometry. While the sample was tilted to a “two-beam” geometry for this experiments, it has been shown that the extraction of absolute values is more rigorous when the sample is tilted to a “three-beam” geometry and the so-called “double-difference” method is utilized^20^,^32^. However, the “three-beam” geometry requires a minimum of four independent scans over the same region, not including the on-axis and low-loss scans. Each additional scan brings a risk of beam damage and dynamic oxidation effects due to the proximity of the iron film to the MgO substrate. In designing this experiment, we made every attempt to avoid such effects, and decided that a controlled systematic error in the absolute values was an acceptable trade-off to reduce the risk of beam damage. This consideration also motivated the choice of aperture position, which was centered on the Thales circle and is the second source of systematic error. Centering the aperture on the Thales circle allows for a significantly higher intensity in the collected EELS spectra and, thus, faster scan rate. However, 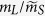 is known to be influenced by the presence of cladding oxide layers^22^ in this aperture position, raising the 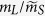 values over the entire dataset. Critically, both of these systematic errors act to translate all of the 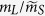 values up or down in concert. We do not anticipate them to selectively influence the relative 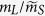 increase observed at the interface, which is the primary finding in this work. Hence, while more work will need to be done to improve our confidence in the absolute values, the relative increase is likely to be physically meaningful.

In summary, we have investigated spin and orbital magnetism in real-space with sub-nanometer spatial resolution at a buried interface of Fe/MgO. Structurally, we find the interface to be free of secondary phases, but the surface roughness of the MgO yields steps so that projection effects lead to a widening of chemical profiles taken over interface. EELS investigations reveal that iron in the proximity of the interface has a change in the white-line ratio and find no evidence for chemical intermixing beyond the broadening of the electron probe and interfacial projection. EMCD measurements reveal a strong increase in 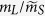 directly at the interface. DFT(+DMFT) simulations predict such an effect for an atomically sharp interface, albeit with a magnitude smaller than the observed effect. We discuss a number of potential sources for error with this experiment, noting that a likely explanation is that a part of the 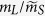 increase at the interface is magnetic in origin. This leads us to conclude that this method has the potential to spatially isolate interfacial phenomena that are related to magnetism with sub-nanometer resolution, extending studies performed with polarized x-rays to the length scales from which spin and orbital magnetism can be directly manipulated.

## Methods

A 50 nm thin film of bcc iron was grown on an MgO substrate using ultra-high vacuum sputtering and prepared for the TEM by polishing, dimple grinding, and bombarding with argon ions. A detailed assessment of the structural quality of this sample can be found in the supplementary information of Thersleff *et al*.^22^. The chemistry of this interface was investigated using core-loss STEM-EELS, and the results of this are presented in the supplementary information. No evidence for Mg interdiffusion beyond the spreading of the signal due to the rough interface was observed. This STEM-EELS experiment as well as the high resolution STEM investigations from Fig. 1 was carried out on a probe-side *C*_*s*_-corrected TEM by JEOL company located at Nagoya University, Japan operated at 200 kV. The EELS data from Fig. 2 and EMCD data from Fig. 3 were acquired on a Tecnai F30 by FEI company equipped with a Tridiem GIF at Uppsala University, Sweden. The TEM was operated at 300 kV in Scanning TEM (STEM) microdiffraction mode, as first proposed by Schattschneider *et al*.^21^ and experimentally expanded in Thersleff *et al*.^22^. The sample was tilted to a two-beam condition with Fe (0 0 2) strongly excited, and the thickness of this region varied between 0.4 and 0.7 *t*/*λ* as estimated from low loss EELS^17^. The thickness map from the region examined here is presented in the supplementary information. This geometry allows for EMCD experiments while keeping the interface parallel to the electron beam. A convergence angle of 2.5 mrad and a collection angle of 3.2 mrad were used, and the collection aperture was placed exactly on the Thales circle. Disregarding beam spreading in the sample, we estimate that the spatial resolution of this configuration approaches 0.8 nm, and this was the pixel sampling size that was chosen. Details on the estimate of this value are provided in the supplementary information. Two individual scans denoted “Chiral Plus” and “Chiral Minus” were acquired this way using the High Angle Annular Dark Field (HAADF) image presented in Fig. 3 as a reference with which the probe position could be corrected between scans. An energy dispersion of 0.2 eV/pixel was used to monitor both the O *K* and Fe *L* transitions, and the energy resolution of these settings was about 1.1 eV. A short dwell time of 0.2 s was used to mitigate the risk of beam damage, and images were acquired before and after the scans to confirm this (see the supplementary information). For each datacube, 6000 individual spectra were acquired with encoded spatial coordinates. Chiral Plus was scanned first, followed by Chiral Minus. Subsequently, the transmitted beam was shifted back into the center of the entrance aperture and the core-loss region was again acquired. Finally, the dispersion was changed to 0.05 eV/pixel to avoid saturation of the CCD camera and the on-axis low-loss region was acquired. A high quality dark reference image of 232 blank spectra was acquired and subtracted individually for each datacube^33^.

The pre-treatment of the EMCD datacubes involves first removing the camera bias observed between quadrants by using principal component analysis (PCA) to observe the components where it has high prominence and then shifting the right 1024 pixels to match. Following this, all components are used to reconstruct the data, retaining their original noise characteristics. X-ray spikes with a variance of greater than^4^ sigma were replaced by an interpolation over the neighboring channel data and plural scattering effects were removed from the individual datacubes by deconvolving them with the low-loss spectra using a zero-loss modifier reconvolution method^17^. The energy drift is accounted for through use of a cross-correlation algorithm over the Fe *L*_3_ edge as implemented in the Digital Micrograph software package. This results in limited gain averaging, further suppressing correlated noise^34^.

The real-space EMCD map from Fig. 3 was generated by using robust PCA^24^ on each individual datacube to extract the first^10^ principal components. Inspection of the scree plot (see supplementary) indicates that a reasonable approximation of the raw data can be achieved with the use of the first four components. The component and score maps from both datacubes are provided in the supplementary information. Each datacube was then reconstructed by using the first 1 to 10 principal components. Following this stage, the pre-edge background for each pixel in each datacube between the energy range 655–690 eV was modeled using an inverse power-law fit implemented in Matlab, extrapolated over the entire energy range, and subtracted^17^. The Chiral Plus datacube was then normalized to the Chiral Minus datacube in the post-edge region between 745–800 eV. The two datacubes from each reconstruction were then subtracted from each other to yield the EMCD spectra and sum rules were applied to the resultant datacube on a pixel-by-pixel basis. Maps for all of the component reconstructions are provided in the supplementary information.

The simulations presented in Fig. 4 were carried out using the Full-Potential Linearized Augmented Plane-Wave (FP-LAPW) method as implemented in the WIEN2k software package^35^ for both the initial optimization of the structure and the subsequent calculations of magnetic properties. Muffin-tin radii were 1.97 a.u. for Mg (1 a.u. = 0.529178 Å), 1.86 a.u. for O, and 2.09 a.u. for Fe. The Perdew, Burke, Ernzerhof form^36^ of the exchange-correlation potential was selected. The plane wave cut-off parameter RK_max_ was set to 7. Relativistic effects were included with the second variational treatment of spin-orbit coupling. The total energy convergence criterion was set to 10^−7^ Ry. In the irreducible wedge of the Brillouin zone, 342 **k**-points were used (35 × 35 × 3 mesh).

The DFT+DMFT calculations were performed using the full-potential linear muffin-tin orbital method as implemented in RSPt code^37^ on top of the L(S)DA exchange correlation functional, using a fully relativistic treatment. We chose the following kinetic energy tails for the basis functions: 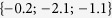 Ry, 32 × 32 × 4 **k**-point grid, and muffin tin radii of 2.1, 1.8, and 2.2 a.u. for Mg, O, and Fe, respectively. We employed the spin-polarized T-matrix + Fluctuating exchange (SPTF)^38^ DMFT solver. Hubbard *U* parameters for Fe 3*d* states were chosen to be layer-independent. Their values were set to *U* = 2.3 eV and *J* = 0.9 eV, and were obtained for bulk bcc Fe from constrained LDA calculations^39^ and employed in several prior DFT+DMFT studies^40^,^41^. The static part of the self-energy was used as a double-counting correction.

## Additional Information

**How to cite this article:** Thersleff, T. *et al*. Towards sub-nanometer real-space observation of spin and orbital magnetism at the Fe/MgO interface. *Sci. Rep.* **7**, 44802; doi: 10.1038/srep44802 (2017).

**Publisher's note:** Springer Nature remains neutral with regard to jurisdictional claims in published maps and institutional affiliations.

## Supplementary Material

Supplementary Information
