# Supplementary material for: Towards sub-nanometer real-space observation of spin and orbital magnetism at the Fe/MgO interface
Source: Sci Rep. 2017 Mar 24;7:44802. doi: 10.1038/srep44802 (PMC5364495; doi:10.1038/srep44802)
Supplement: Supplementary Information [file srep44802-s1.pdf]

Supplementary Information: Towards sub-nanometer real-space observation of spin and orbital magnetism at the Fe / MgO interface

Thomas Thersleff,[1,*] Shunsuke Muto,[2] Mirosław Werwiński,[3,4] Jakob Spiegelberg,[3] Yaroslav Kvashnin,[3] Björgvin Hjörvarsson,[3] Olle Eriksson,[3] Ján Rusz,[3] and Klaus Leifer[1]

(STINT Collaboration)

[1]*Department of Engineering Sciences, Uppsala University, Box 534, 75121 Uppsala, Sweden*

[2]*Advanced Measurement Technology Center, Institute of Materials and Systems for Sustainability Chikusa-ku, 464-8603 Nagoya, Japan*

[3]*Department of Physics and Astronomy, Uppsala University, Box 516, 75120 Uppsala, Sweden*

[4]*Institute of Molecular Physics Polish Academy of Sciences, M. Smoluchowskiego 17, 60-179 Poznań, Poland*

(Dated: February 10, 2017)

ADDITIONAL HRTEM DATA FROM THE INTERFACE

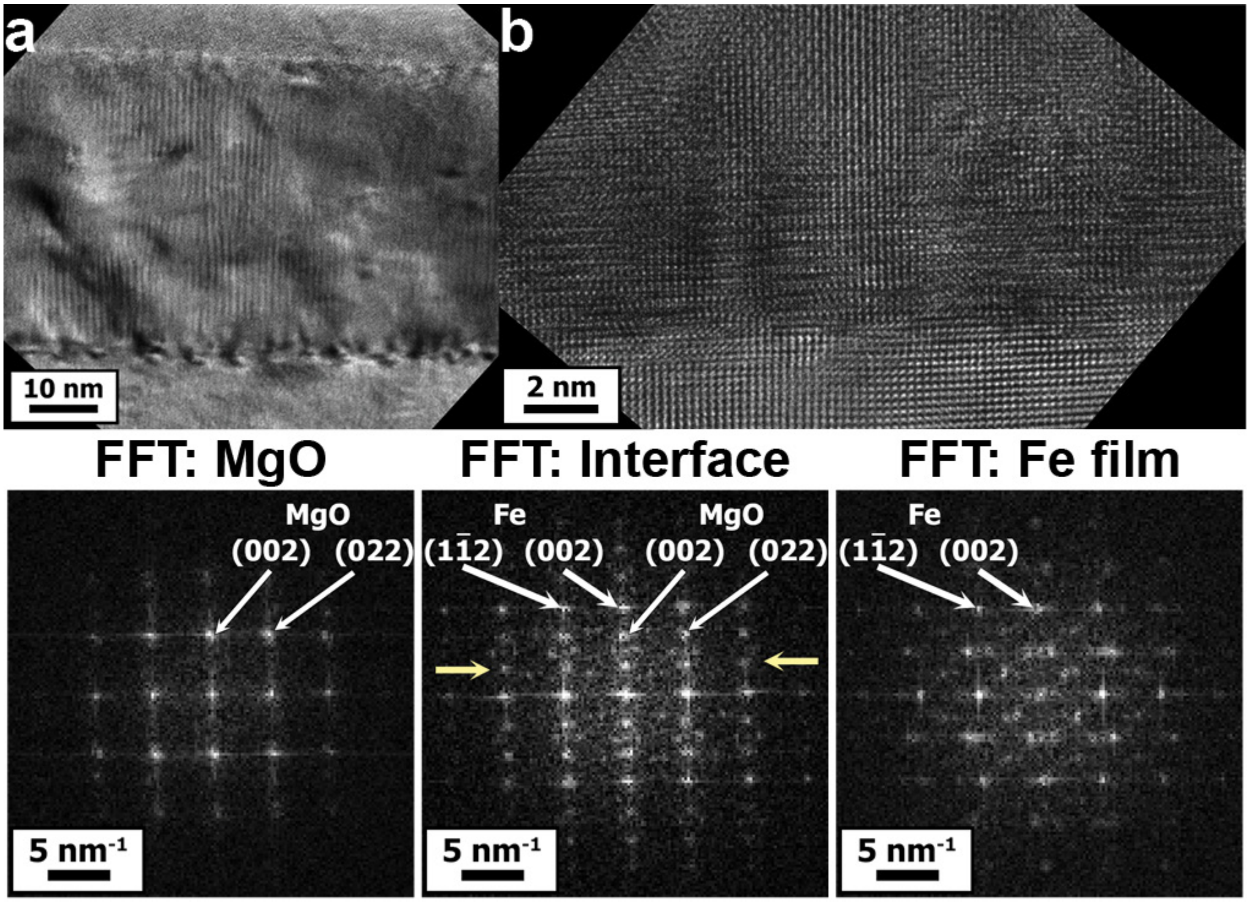

FIG. 1. (a) Two-beam condition using $\mathbf{g} = (1\ 1\ 0)_{\mathrm{Fe}}$. (b) HRTEM of the interface. A strong Moiré contrast is observed. Below, Fourier transforms of the MgO, interface, and Fe regions are presented. Yellow arrows point to the spatial frequencies responsible for the observed Moiré fringes.

The structure of the Fe/MgO interface grown for this study was investigated through the use of HRTEM, and the results of this are shown in figure 1. In figure 1a, the sample was tilted to a two-beam condition with $\mathbf{g} = (1\ 1\ 0)_{\mathrm{Fe}}$. Vertical streaks within the iron film are the consequence of a Moiré contrast generated between the cladding oxide layers and the metallic underlayer, and is discussed in greater detail in the supplementary information of [1]. At the interface, however, an alternating contrast bowing upwards into the Fe film can be observed. This is likely due to local alterations of the crystallographic symmetry due to misfit dislocations in this region. The spacing of these misfit dislocations is approximately 4 nm, consistent with previous reports [2]. In figure 1b, a HRTEM micrograph of the interface

is presented. The defocus value in this image is close to Gaussian defocus. Below, the modulus of three Fourier transforms from the regions denoted are presented. The presence of MgO and metallic iron can be inferred from the reflections visible in the substrate and film regions, respectively. However, the Fourier transform from the interfacial region (denoted as the region with an additional horizontal frequency) appears to contain both Fe and MgO reflections. Moreover, an additional reflection, denoted with the yellow arrows, appears in the FFT. This reflection fits well with the kinematically-forbidden MgO (0 0 1) reflection, and has been observed previously as well [3]. It is suspected that this arises from surface roughness on the MgO and the three-dimensional growth of the subsequent Fe layer, which is thus offset by half of an MgO unit cell in the growth direction for several nanometers [4].

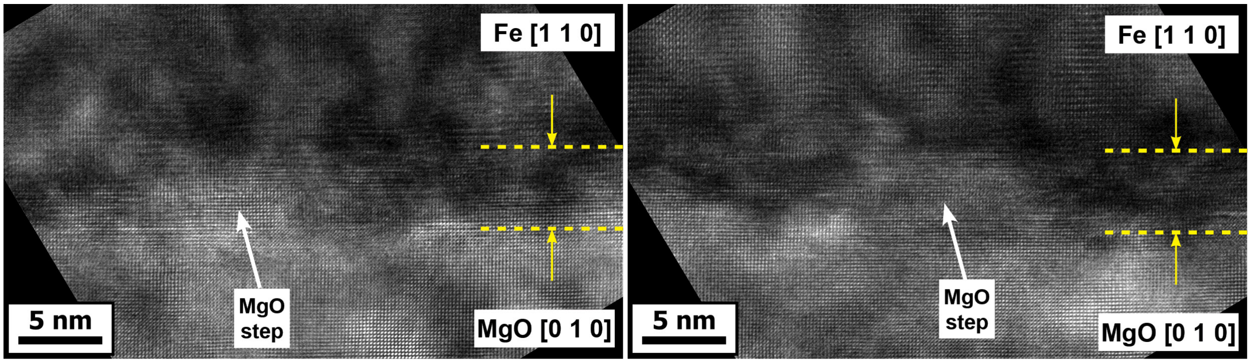

FIG. 2. Two HRTEM micrographs from different sample regions revealing large surface steps in the MgO substrate that propagate into the iron thin film region. The lateral distance over which the interface is suspected to be projected is estimated to lie between the dashed yellow lines.

Direct observations of such steps are presented in figure 2. Two independent regions of the sample were investigated where perturbations of the MgO surface were suspected to arise. These HRTEM images were acquired on a C_S-corrected ARM TEM (JEOL company) at Nagoya University, Nagoya, Japan. The steps disturb the subsequent iron growth along the direction of the electron beam propagation, leading to the Moiré contrast observed in figure 1b. The height of the steps can be estimated in these images since the MgO step appears to have extended through most of the TEM lamella in this region. We observe that the disturbed region is on the order of 5 nm, suggesting that the projection of the interface also extends over this distance for the EMCD experiments. Significantly, these HRTEM investigations reveal no clear evidence for the formation of a secondary phase at this interface, further supporting the interpretation that the oxide signal observed in the

EELS data comes from the direct bond between the bulk iron atoms and the oxygen atoms within the MgO substrate itself. Hence, we conclude that the interfacially-bonded iron atoms are projected over a length of some nanometers, resulting in what appears to be an extended interfacial region, similar to the conclusions of Serin et al. [3].

CHEMICAL PROFILE OF THE INTERFACE

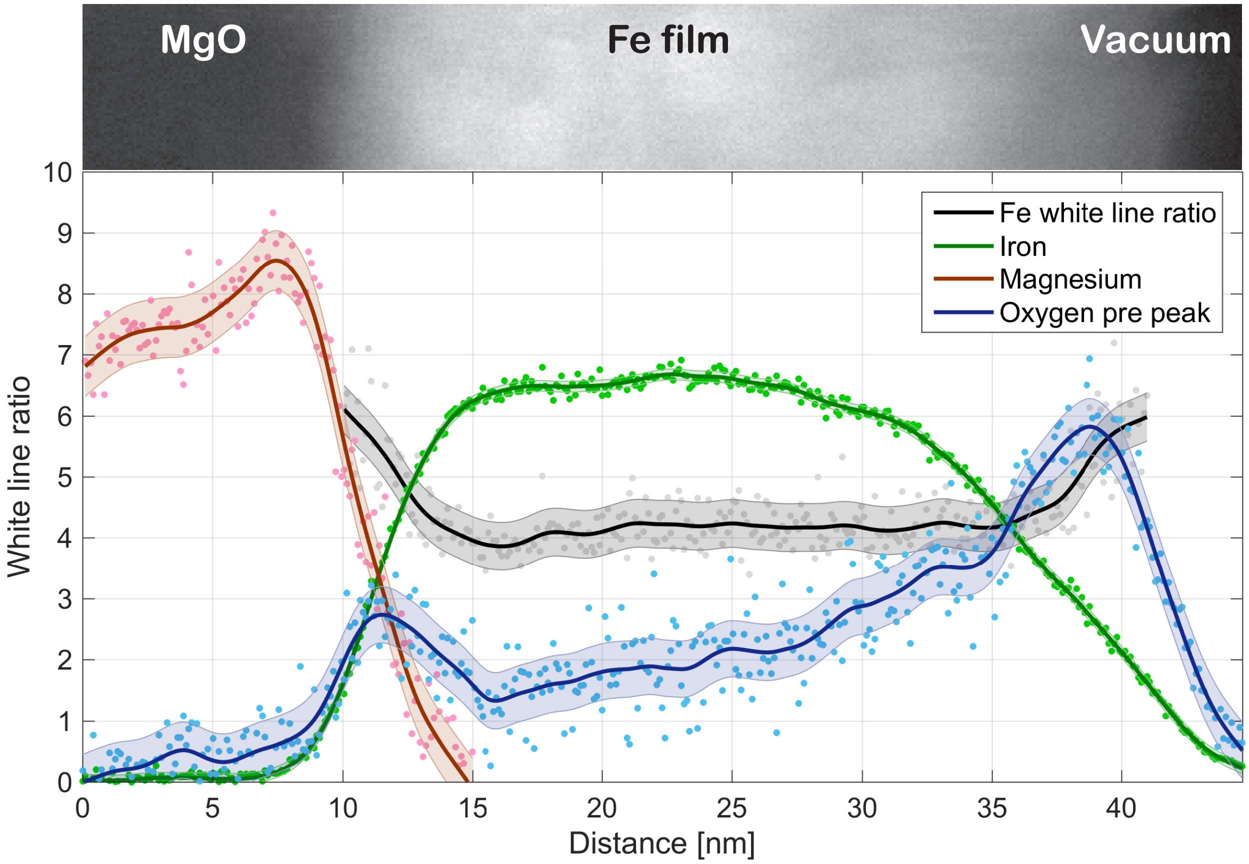

FIG. 3. Chemical analysis of the interface investigated with EELS. At top, the HAADF survey image is presented on the same x-axis as the line profiles below. The line profiles of the integrated intensities of the oxygen pre-peak, the iron post-edge region, and the Magnesium post-edge region are plotted in blue, green, and red, respectively. These are scaled relative to each other. The white-line ratio of the Fe L_3 / L_2 edges is plotted in black and the values are presented on the y-axis to the left. The raw data for all of these curves is plotted as closed circles with a bold line representing a spline fit and the one sigma confidence interval is shown as a translucent overlay. A clear increase in the white-line ratio is observed within 5 nm of the interface

The chemistry of the interface from a different region was investigated using EELS on a probe C_S corrected TEM (JEOL, Inc.) at Nagoya University, Japan. At the top of figure 3, the HAADF survey image is provided and the vacuum, film, and MgO regions are labeled. A spectrum image from this region was acquired and the resulting datacube was vertically summed into a line scan across the region shown. The integrated intensities of the iron and magnesium post edge regions are plotted on the same x-axis as the HAADF image. For the oxygen profile, the pre-peak was integrated rather than the post-edge region allowing for a qualitative segregation of iron oxide from the oxygen in the MgO substrate [5]. These profiles are scaled to fit a common range to visualize the qualitative trends across the bilayer. The white-line ratio of the iron was extracted using the technique outlined in [3, 6] and is presented in absolute units appearing on the y-axis.

The reduction of the Mg signal provides some insight into the extent of the projection of the atomic steps into the iron layer as well as the influence of beam broadening due to the large convergence angle (30 mrad), similar to prior studies showing similar dechanneling behavior [2, 3]. Beyond this broadening, there is no evidence for interdiffusion of Mg. Over the same length scale, a strong increase in the iron white-line ratio is observed, once again consistent with previous investigations [2, 3]. The white line ratio change is known to be related to an increase in the oxidation state of iron [5] and is accompanied by an increase in the intensity of the oxygen pre-peak, clearly indicating that the iron in the interfacial proximity is bonded with oxygen in some manner.

PRESENTATION OF THE RAW EMCD SPECTRA

The raw EMCD data are presented in figure 4. The pre-treatment steps described in the methods section of the manuscript were applied to these data; they otherwise were untreated before this stage. The spectra here were generated by summing the data over two different regions: the entire spectrum image (full) and the three columns at the interface (interface). This technique has been shown in a previous study to dramatically improve the signal to noise ratio of the EMCD spectra, at the cost of reduced spatial resolution [1]. The raw, untreated spectra are shown for these two summations in figure 4a and d. The pre-edge background model and its extrapolation is also presented. This background was subsequently subtracted and the spectra were normalized to the post-edge region shown in

figure 4b and e. At bottom of each of these graphs, the difference between the two spectra is shown. This is the EMCD signal of interest. The y-axis is normalized to the maximum value of the Chiral Minus spectrum in each case, and the strength of the EMCD signal approaches 6%.

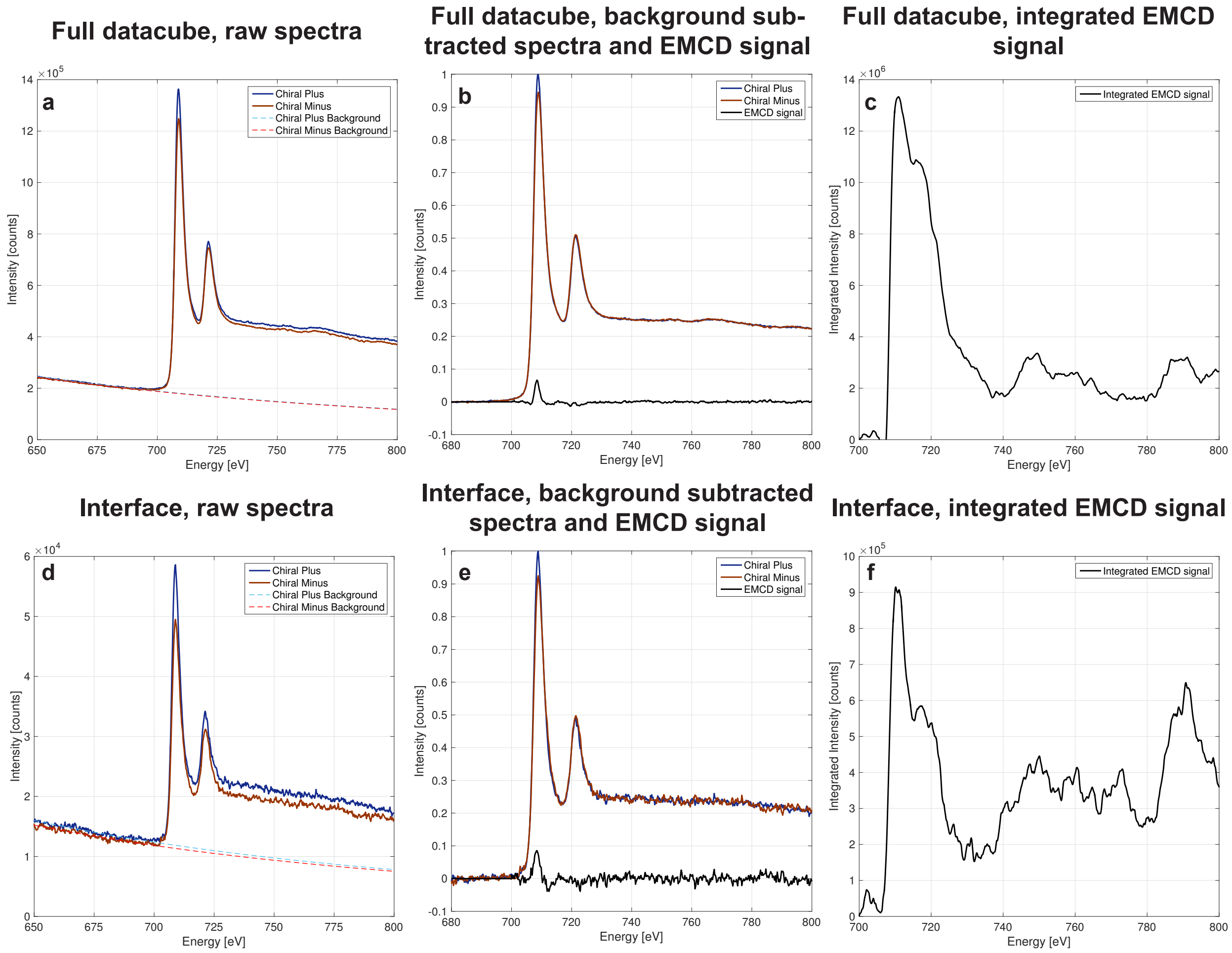

FIG. 4. Summation of all raw spectra in both the Chiral Plus and Chiral Minus spectrum images for the full datacube (a) and the interface (d). The pre-edge background for both spectra is presented along with its extrapolation under the iron ionization edges. The background subtracted spectra after post-edge normalization along with the EMCD signal is presented in (b) for the full datacube and (e) for the interface. The integral of the EMCD signal is presented in (c) for the full datacube and (f) for the interface.

The EMCD signal is subsequently integrated and the result of this operation is presented in figure 4c and f. A few anomolies are observed in the post-edge region, and these are accounted for by using a very large post-edge normalization window (see the supplementary

information in Thersleff et al. [1]). Application of sum rules to these spectra and propagation of error yields $m_L/m_S = 0.07 \pm 0.02$ for the full summation and $m_L/m_S = 0.22 \pm 0.15$ for the interface. The value for the full summation is slightly higher than what would be expected for bulk iron, which is consistent with the position of the collection aperture on the exact Thales circle as well as with the use of a large collection angle, as described in Thersleff et al. [1]. The enhancement of $m_L/\tilde{m}_S$ is thus observed even in the raw data, albeit with greatly reduced confidence.

EXPERIMENTAL GEOMETRY

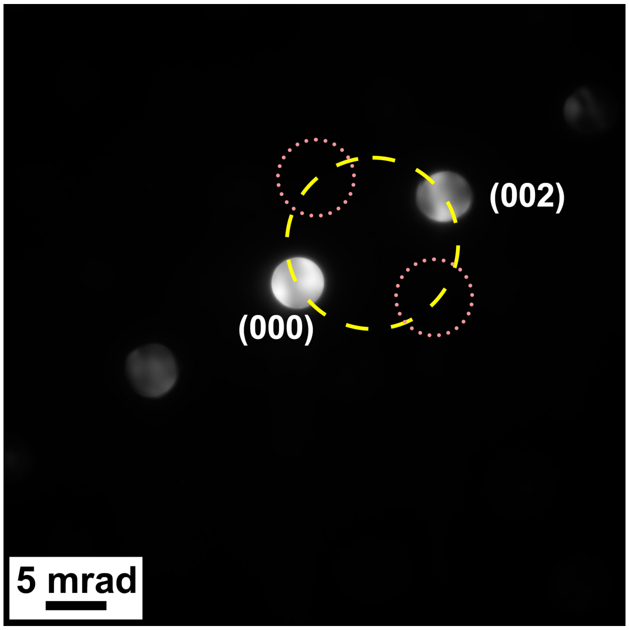

FIG. 5. CBED pattern from the iron lamella in the experimental geometry used for the EMCD experiment. The Thales circle is marked as a dashed yellow line while the position of the collection apertures is marked with a dotted pink outline.

The experimental geometry for the EMCD investigation is presented in figure 5. The sample was tilted to a two-beam orientation with the Fe (0 0 2) reflection strongly excited. The Thales circle for this configuration is presented in figure 5 as a dashed yellow outline. The collection apertures for the Chiral Plus and Chiral Minus scans were placed in the positions denoted by the dotted pink outlines. This pattern was acquired shortly before the EMCD scans were executed.

LAMELLA THICKNESS

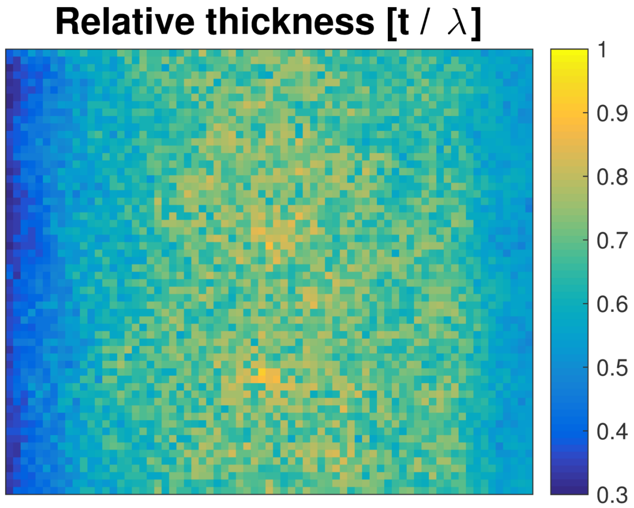

FIG. 6. Relative thickness of the area scanned in the EMCD experiment. Values are provided in units of t/λ where t is the absolute thickness and λ is the electron mean free path.

Since the low-loss spectra for this lamella were recorded to allow for the removal of plural scattering events from the EMCD data, it is also possible to calculate the thickness of the lamella on a pixel-by-pixel basis. This is performed in figure 6. Throughout the bulk of the lamella, the thickness remains relatively constant at around 0.68 ± 0.06 mean free paths. The reduction on the right side of the image is partially caused by the overlap of the MgO interface with the Fe thin film. A calculation of the absolute thickness of the iron layer is complicated by this overlap, which takes place together with the cladding oxide layers. The thickness of these oxide layers can only be determined in combination with a structural investigation that yields a reasonable assumption for the structure and density of the oxide layers.

OXYGEN PRE-PEAK INTEGRATION WINDOW

Key to understanding the chemical behavior at the interface is the observation of the oxygen K-edge in the EELS data. This edge is well documented for both iron oxide compounds as well as for MgO. The oxygen pre-peak is observed at an energy loss that is significantly lower than the oxygen edge for MgO, providing a way to segregate the two signals. This allows for a statement about the presence of oxidized iron at the interface, be it either due to

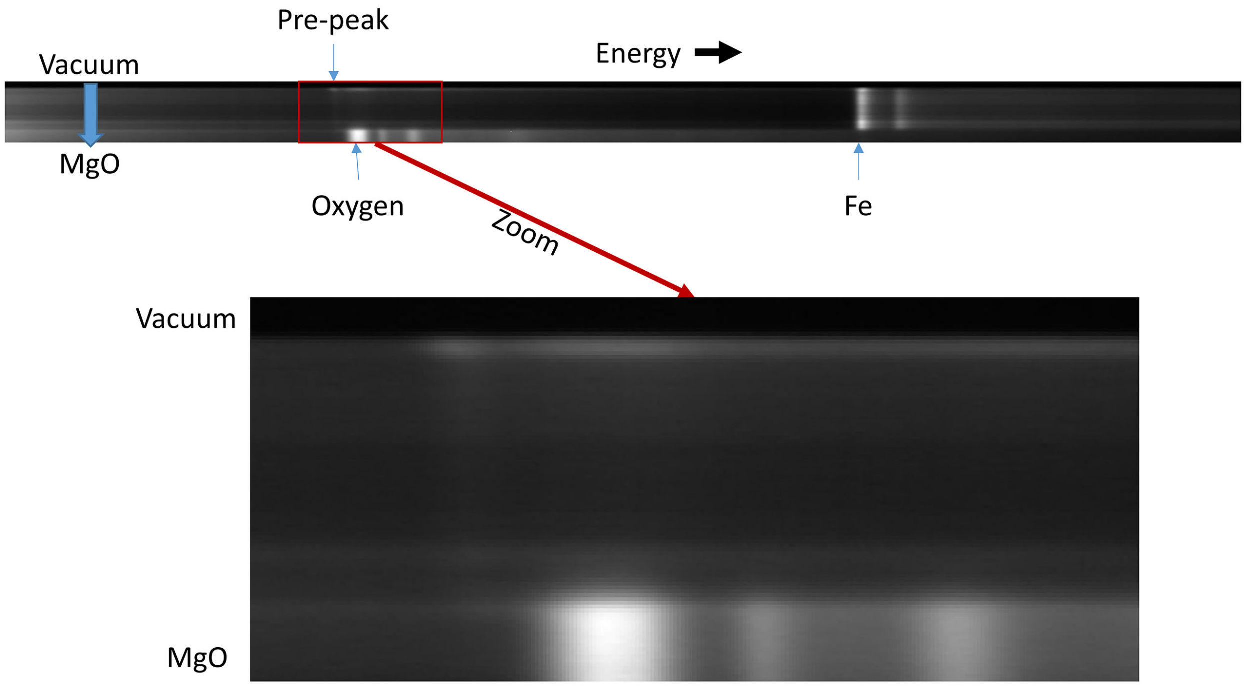

FIG. 7. At top, the line profile for the EELS data following summation along the interface parallel direction is presented. The vacuum is near the top while the MgO substrate appears at the bottom. A close-up of the oxygen edge is presented below and the positions of five spectra analyzed in figure 8 are denoted.

the formation of a reaction layer or the actual interfacial bond. To investigate this, we used the EELS datacube acquired with the collection aperture centered around the transmitted beam. This datacube was acquired from the same area as the EMCD experiment and the pre-treatment was the same, including energy drift correction using the iron L_3 edge, as described in the manuscript. Following this stage, the datacube was summed along the direction parallel to the interface to yield a line scan across it. The result of this summation is presented in figure 7.

Five spectra from this line scan were extracted for closer inspection. These spectra represent regions of interest corresponding to the MgO substrate, the surface oxide layer, the bulk iron, the region structurally disrupted by MgO surface steps (denoted “Moiré” here, see figure 2 for a secondary reference), and finally the spectra corresponding to the Fe/MgO interface. These five spectra are plotted together in figure 8a, and scaled for better visibility of the pre-peak in figure 8b. The clear energy separation between the oxygen

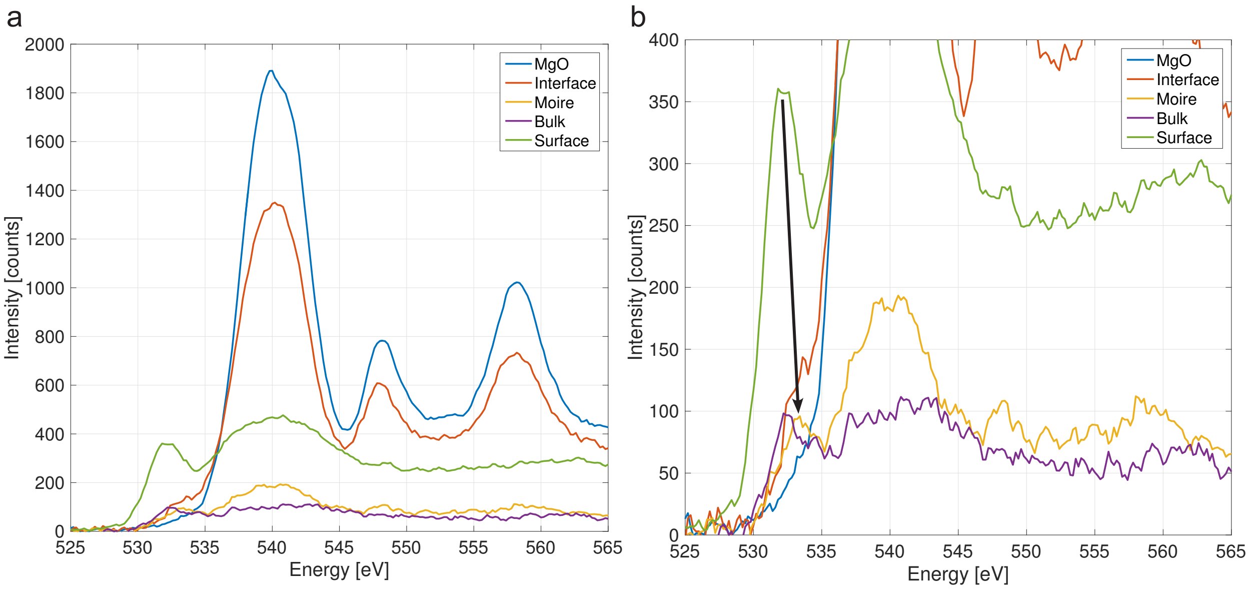

FIG. 8. (a) The raw data for the oxygen pre-peak, plotted after background subtraction. The data are summed over columns as denoted in the figure legend. (b) Same data as in (a), but scaled to magnify the pre-peak positions. A clear chemical shift in the oxygen pre-peak is observed between the surface and the interface.

edge from the MgO and the iron oxide is apparent and is sufficiently large for this analysis to distinguish. An additional important observation is that the onset position of the pre-peak appears to shift to higher energy loss close to the interface. This is most apparent in the "Moiré" spectrum but is also evident at the interface, where the pre-peak appears as a shoulder superimposed over the MgO edge. This shift cannot be explained by instrumental energy drift, as all spectra were aligned to the Fe L_3 edge. Moreover, each one of these spectra represents a summation over 60 individual spectra. Hence the energy shift must be a physically relevant effect related to chemical shifts.

The shifting of the oxygen pre-peak complicates the placement of the integration window used to track concentration gradients over the interface. Too large of a window includes the oxygen signal from the MgO, but smaller windows yield noisier data. Also, if the window is placed at too high of an energy, the MgO signal becomes dominant near the interface. To optimize this process, we chose a window width of 1.0 eV and varied its starting position in 1 eV increments starting at 528 eV. The integration of all the spectra in the line scan for each window position is presented in figure 9. It is immediately clear that, when the window

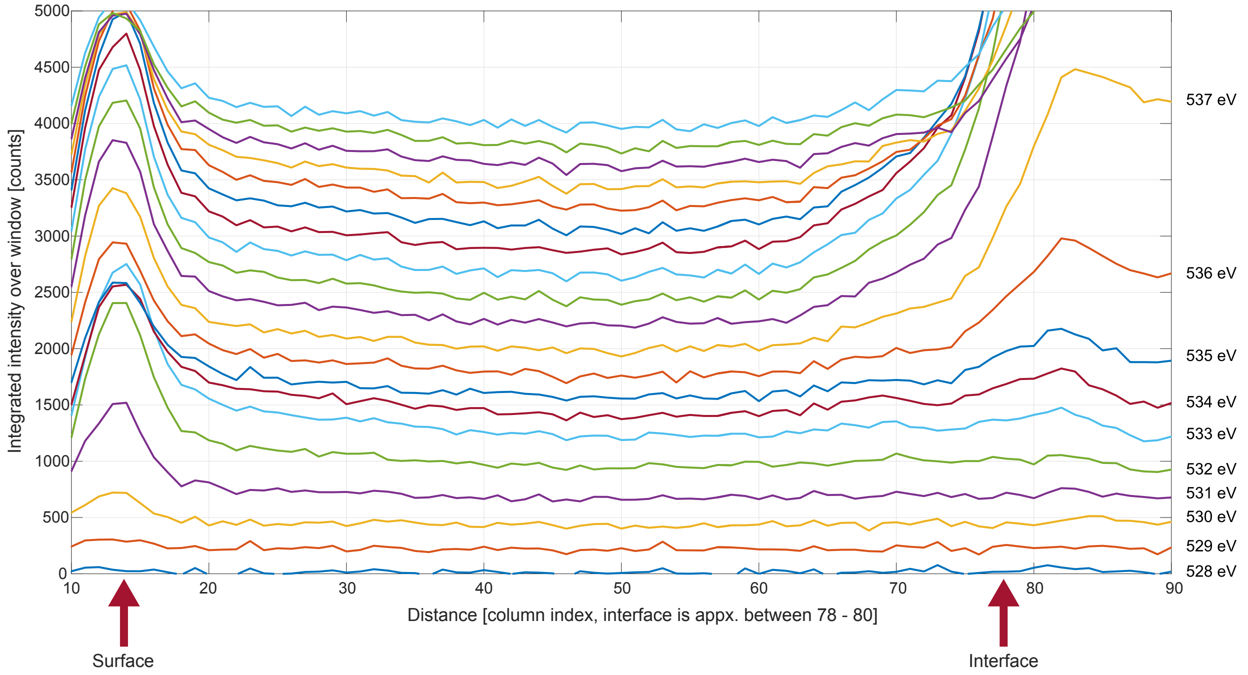

FIG. 9. Watershed plot of the oxygen pre-peak concentration profile mapped perpendicular to the interface as a function of the position of the integration window, which is denoted at right. The window width was kept constant at 1.0 eV for each profile.

is placed close to the onset of the MgO oxygen edge, then a large increase in the oxygen concentration is observed that is unlikely to be caused by the presence of an iron oxide. This increase extends away from the interface into the iron film by a significant amount and may lead to the false conclusion that there is an additional oxide layer present at the interface. However, if the window is placed at a lower energy loss such as 532 eV, then the pre-peak belonging to iron oxide is successfully segregated from the MgO, and no additional increase of oxygen is observed at the interface. The oxygen pre-peak is known to originate from the contaminating oxide layers that grow on the iron surface, and this result indicates that the amount and, hence, thickness of these layers remains invariant as one moves closer to the Fe / MgO interface.

A more detailed analysis of the oxygen chemistry of the interface was carried out by using multiple linear least-squares (MLLS) fitting of the experimental spectra to reference spectra. Two reference spectra were used for the fitting procedure: the surface oxide and the MgO spectra (see figure 8a). These two reference spectra were then linearly combined

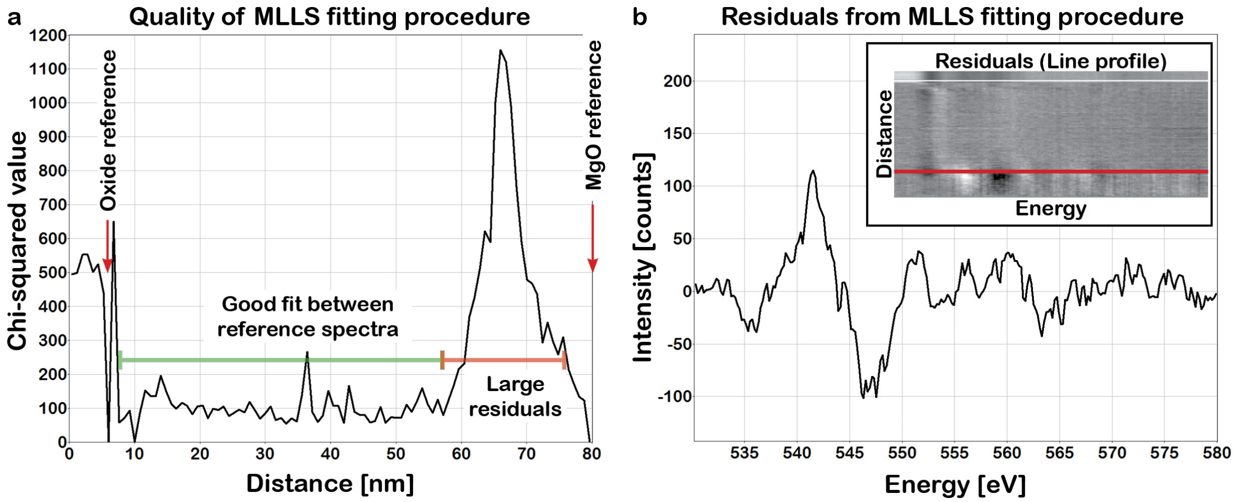

FIG. 10. (a) Results from MLLS fitting routine using the surface oxide and MgO spectra as references. Near the Fe / MgO interface, these two spectra fail to adequately describe the experimentally observed spectra. (b) Residuals from the MLLS fitting procedure on the oxygen edge. The full line scan is presented as a 2D image in the inset, with the red line denoting the displayed spectrum. The white line at the top represents the oxide reference.

to explain the spectral features in the raw data. The results of this analysis are presented in figure 10. In 10a, the chi-squared value of this fitting routine is shown. The spectra from bulk iron as well as the spectra within the MgO are well described by the surface oxide and MgO reference spectra, and the chi-squared value is largely a reflection of residual noise with little covariance. This indicates that the contamination oxide layers are chemically similar to what is found near the vacuum. However, at the interface, these two reference spectra fail to adequately describe the experimental observations, resulting in very large chi-squared values. The residuals are plotted in figure 10b and a line scan across the interface region is shown. Strong residuals peaks are observed (denoted with the arrows). Hence it can be understood that the oxygen spectral shape in this region differs significantly from either MgO or the surface iron oxide. We interpret this to mean that the chemical nature of the oxide interfacial layer is distinct from the surface contamination layers. This would be the case if the iron atoms in the bulk iron film directly bonded to the oxygen atoms in the MgO substrate. Such a configuration would also likely result in a chemical shift of the oxygen pre-peak, and would explain the experimental observations of figure 8. Thus, we find no evidence of an oxide reaction layer that forms at the interface, concluding that the white-line

ratio increase and, subsequently, increase in $m_L/\tilde{m}_S$ arises from the interfacial bond between iron atoms and the oxygen atoms in the MgO substrate.

BEAM DAMAGE ASSESSMENT

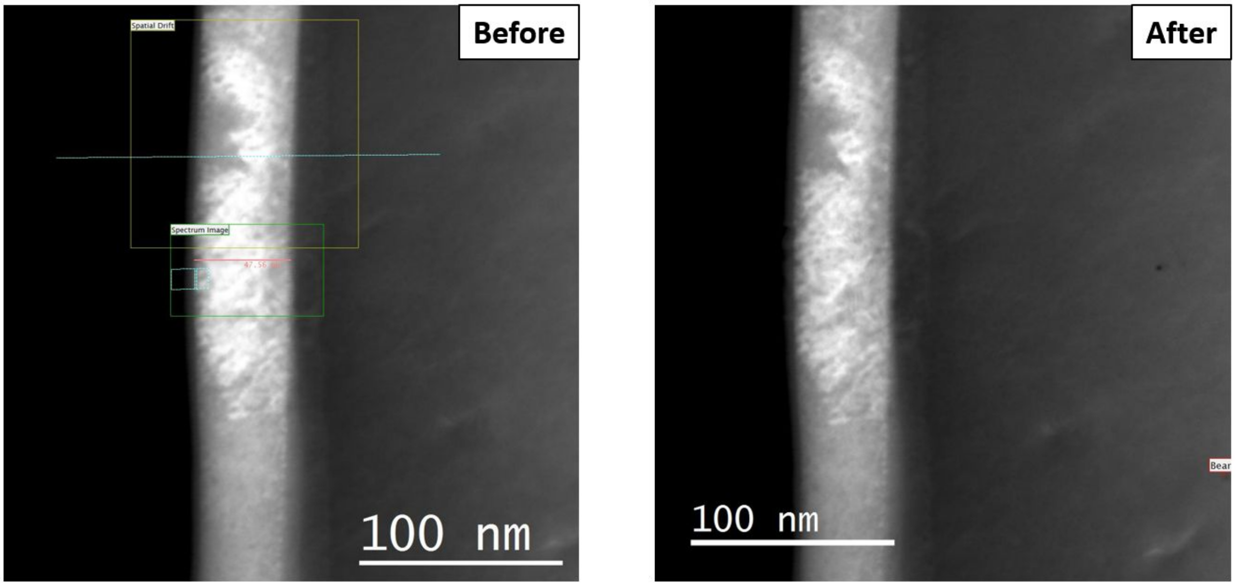

FIG. 11. Two HAADF survey images taken before (left) and after (right) the acquisition of the EMCD datacubes. No strong evidence for modification of either the iron or the MgO substrate due to the electron beam interaction is observed.

The STEM-EMCD experiment presented in the manuscript was designed to minimize beam damage to the sample as best as possible while still retaining a large enough signal to noise ratio in the raw spectra to allow for EMCD analysis. The concern is that the beam would decompose the MgO substrate between the Chiral Plus and Chiral Minus scans, thereby resulting in an artificial increase in the white line ratio that could be misinterpreted as magnetic in origin. We stress here that we cannot completely rule out this effect with the present experimental design, and we have attempted to articulate this in the manuscript. Nevertheless, we have taken the following efforts to minimize the chance that such an effect could take place.

First, the pixel dwell time was minimized to prevent excessive radiation dosage to the sample. This was accomplished by using a pixel dwell time of 0.2 s. The loss in signal was compensated for by collecting a large quantity of spectra (6000 for each datacube) and

increasing the collection angle. This improved the spatial resolution of the experiment but also reduced the angular sensitivity. Our findings indicate that this is an acceptable trade-off to make, provided that the signal to noise level of an individual spectrum is sufficiently large for the variance of the EMCD signal to be captured by the subsequent multivariate statistical treatment.

Second, prior to the scan, raw spectra from neighboring regions were inspected to determine which aperture pairing yielded iron ionization edges with the highest white line ratio. In this case, it was the Chiral Plus pairing (see figure 4). This pairing was then the first one used for the STEM-EMCD experiment. If an in-situ oxidation were to take place, one would expect the white line ratio of the second scan to increase as a result, potentially canceling the magnetic effect. This was not observed; the Chiral Minus datacube has a lower white line ratio than the Chiral Plus datacube.

Third, the intensity of the oxygen pre-peak was monitored for both STEM-EMCD datacubes. These results are presented in figure 2 of the manuscript and show that the oxygen level does not vary with statistical significance between the two scans.

Fourth, the beam was scanned perpendicular to the Fe / MgO interface. If in-situ oxidation were to occur, then it should result in a gradual increase in the white line ratio during the scan. This was not observed, as evidenced by the $m_L/\tilde{m}_S$ maps.

Finally, the survey region was scanned both before and after the data acquisition to inspect for structural and z-contrast differences. These scans are presented in figure 11. Significant variations were neither observed in the iron film nor in the MgO substrate. The small differences in contrast between the two images can be attributed to changes in the electron optical conditions arising from the reduction of the incident beam current necessary to acquire the low-loss spectrum image.

ROBUST PCA ANALYSIS

The raw datacubes were decomposed using robust PCA [7]. The scree plots from this decomposition are presented on a log plot in 12a,c for Chiral Plus and Chiral Minus, respectively. A reasonable description of the original variance of the datacube appears to be achieved after four components. The score outlier maps from this decomposition are presented in figure 12b,d for Chiral Plus and Chiral Minus, respectively. RPCA assures that

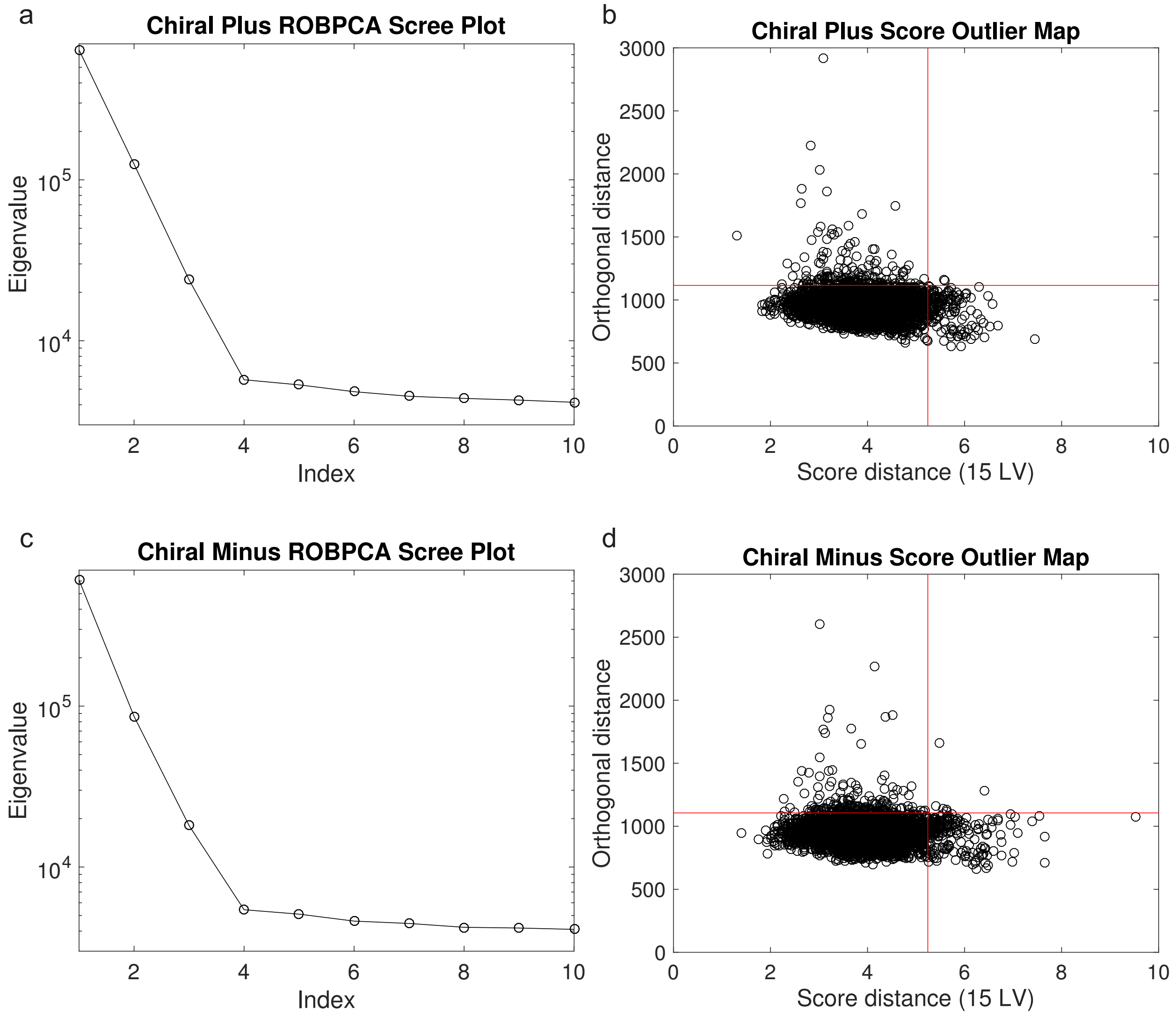

FIG. 12. The scree plot (a) and score outlier map (b) for the Chiral Plus datacube. The scree plot (c) and score outlier map (d) for the Chiral Minus datacube.

the variance of spectra displaying artifacts due to the deconvolution routine is not considered for the estimation of the principal components. The graphs from both datacubes look remarkably similar.

The score maps and loading curves for both the Chiral Plus and Chiral Minus datacubes are presented in figures 13 and 14, respectively. It is immediately clear that the decomposition is nearly identical for both datacubes, with the occasional sign flip being the only large deviation. The first four components in both datacubes are strongly localized in their score maps and have clear spectral signatures. Components number 5 and above no longer ex-

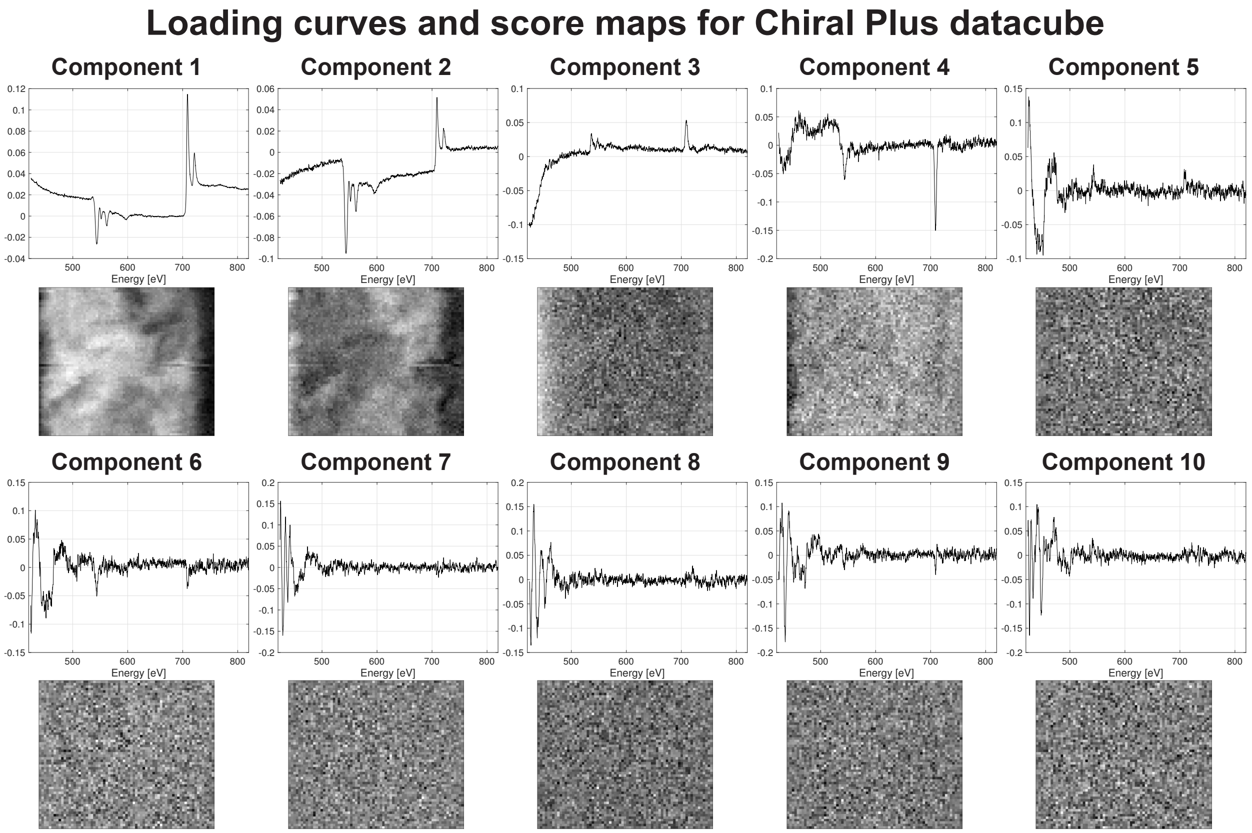

FIG. 13. The score maps and loading curves for the first 10 components of the Chiral Plus datacube.

hibit such strong spatial localization and their spectral signatures have significantly reduced variances, as evidenced by the scree plots in figure 12. Some residual spectral signatures in the iron and oxygen regions are visible for all components; however, the lack of strong spatial localization means that they have little influence on the EMCD signal strength, rather increasing the total noise distribution in the final $m_L/\tilde{m}_S$ maps.

Although there is no strong spatial localization at the interface of the PCA components above number 4, it is still instructive to retain these additional components when generating the real-space $m_L/\tilde{m}_S$ maps. This was done for all components between 2 and 9, as presented in figure 15. The median value for each column is also computed and presented as a line scan across the interface. A clear enhancement of $m_L/\tilde{m}_S$ is observed for all reconstructions using up to 6 components. The noise of the maps also increases accordingly, leading to higher uncertainties when computing the pre-edge background, post-edge normalization, and application of the sum rules. This is a consequence of the lack of spatial localization in PCA

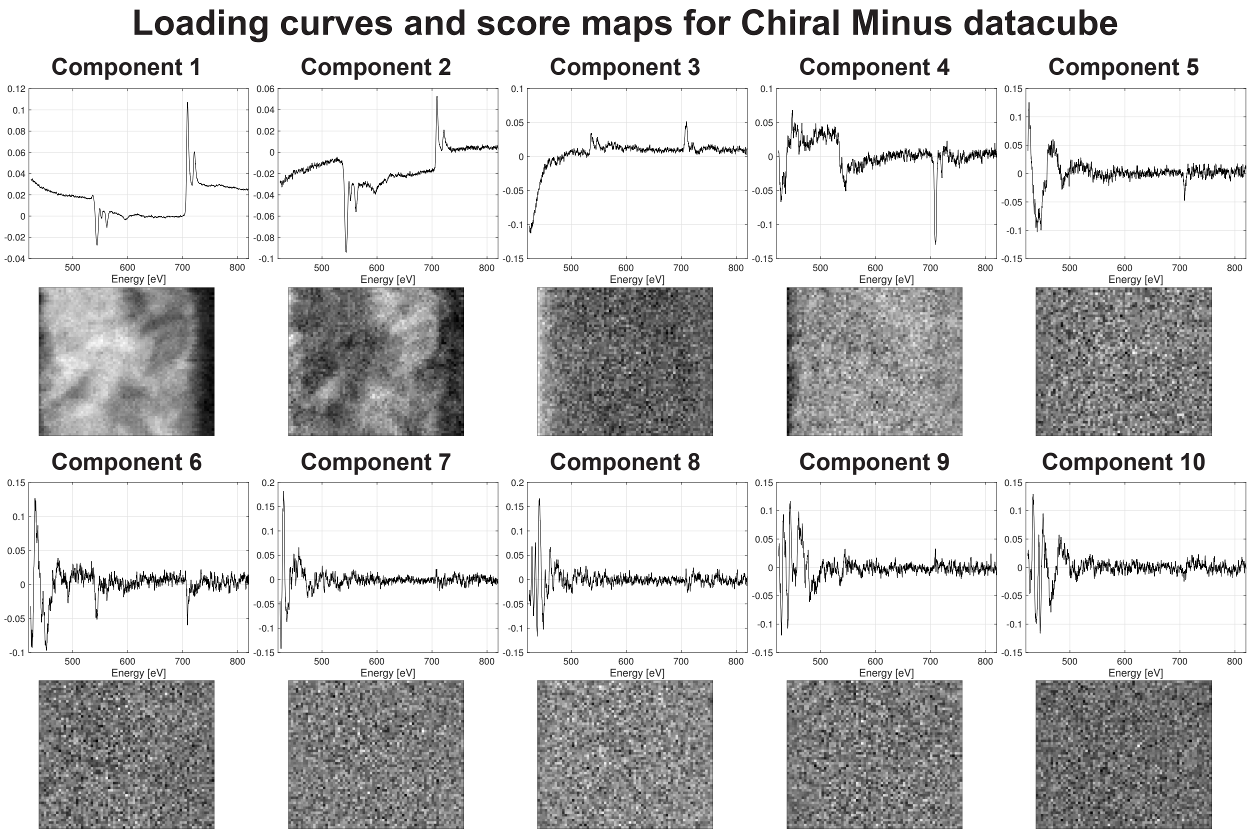

FIG. 14. The score maps and loading curves for the first 10 components of the Chiral Minus datacube.

components above number 4. Thus we can say with high confidence that the physically meaningful components responsible for the enhancement of $m_L/\tilde{m}_S$ at the interface are reliably captured with this method. Any physically meaningful variance not captured within the raw dataset is on the order of magnitude of the noise level and is unlikely to impact the finding of the strongly localized $m_L/\tilde{m}_S$ enhancement at the interface.

ARTIFICIAL SPATIAL SHIFTING OF m_L/m_S MAPS

As discussed in the manuscript, one of the potential sources for systematic error in this experiment is a spatial drift that takes place between scans. The concern is that, if one scan contains a larger percentage of interfacial iron atoms than the second, then some part of the white line ratio change will be due to charge transfer rather than magnetism. This would be difficult to detect and disentangle from a magnetic effect and thus adds uncertainty to

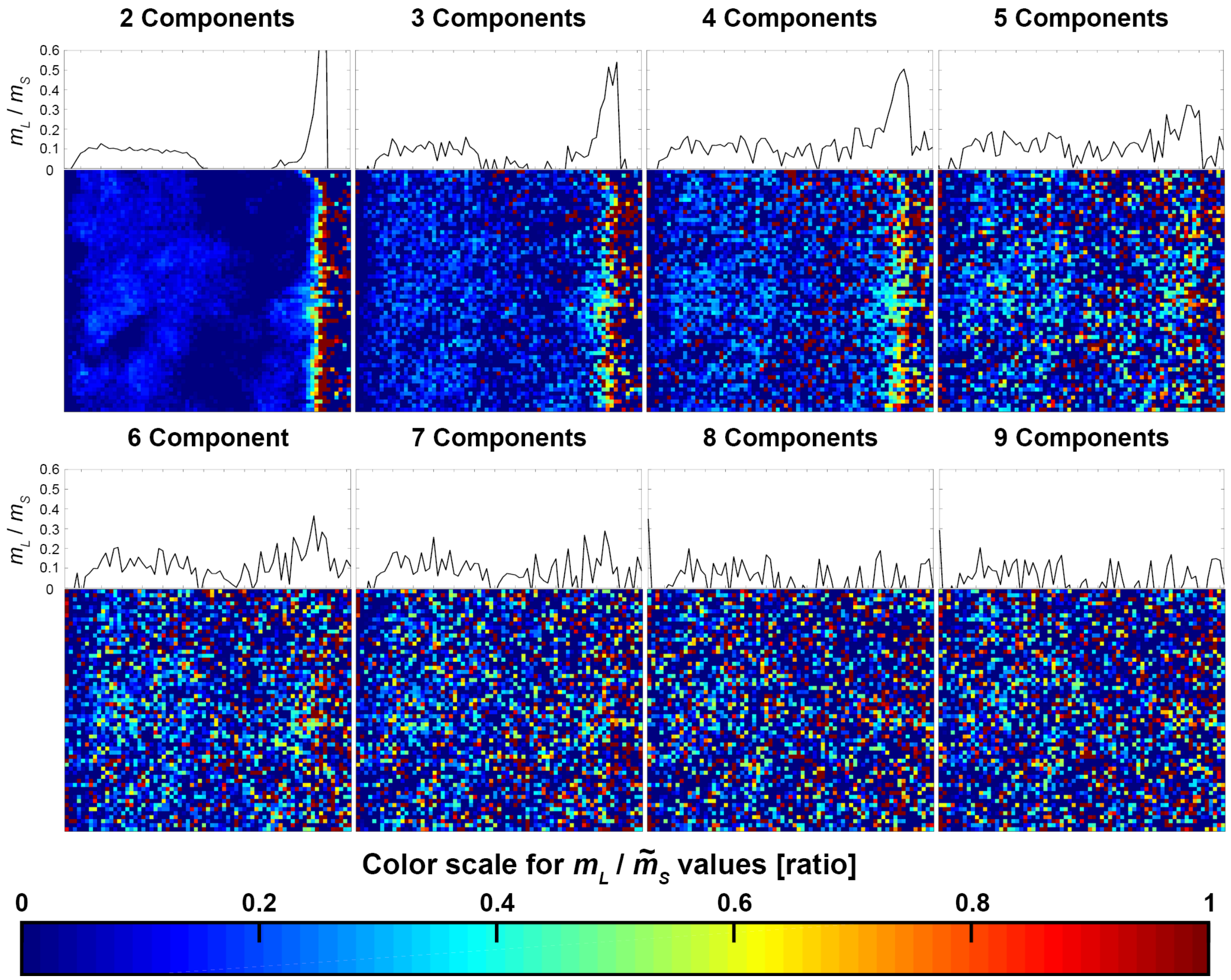

FIG. 15. The $m_L/\tilde{m}_S$ maps for matrix reconstructions using 2 – 9 components. Also presented are line scans of the median $m_L/\tilde{m}_S$ values as a function of distance across the interface.

the results.

To understand this effect, we artificially shifted the two datacubes with respect to one another. The result of this operation is presented in figure 16. These maps were generated by shifting the datacubes relative to each other in the direction perpendicular to the interface by between -4 to +4 pixels before applying the sum rules on a pixel-by-pixel basis. This was performed with the map reconstructions for three, four, and five principal components.

We observe that, for very large shifts, some structure appears in the iron bulk. This likely corresponds to regions of the sample with different oxide contents being compared, and justifies our more general concern that the probe position must be very accurate between scans for a pixel-by-pixel comparison to work. However, we also observe that these additional

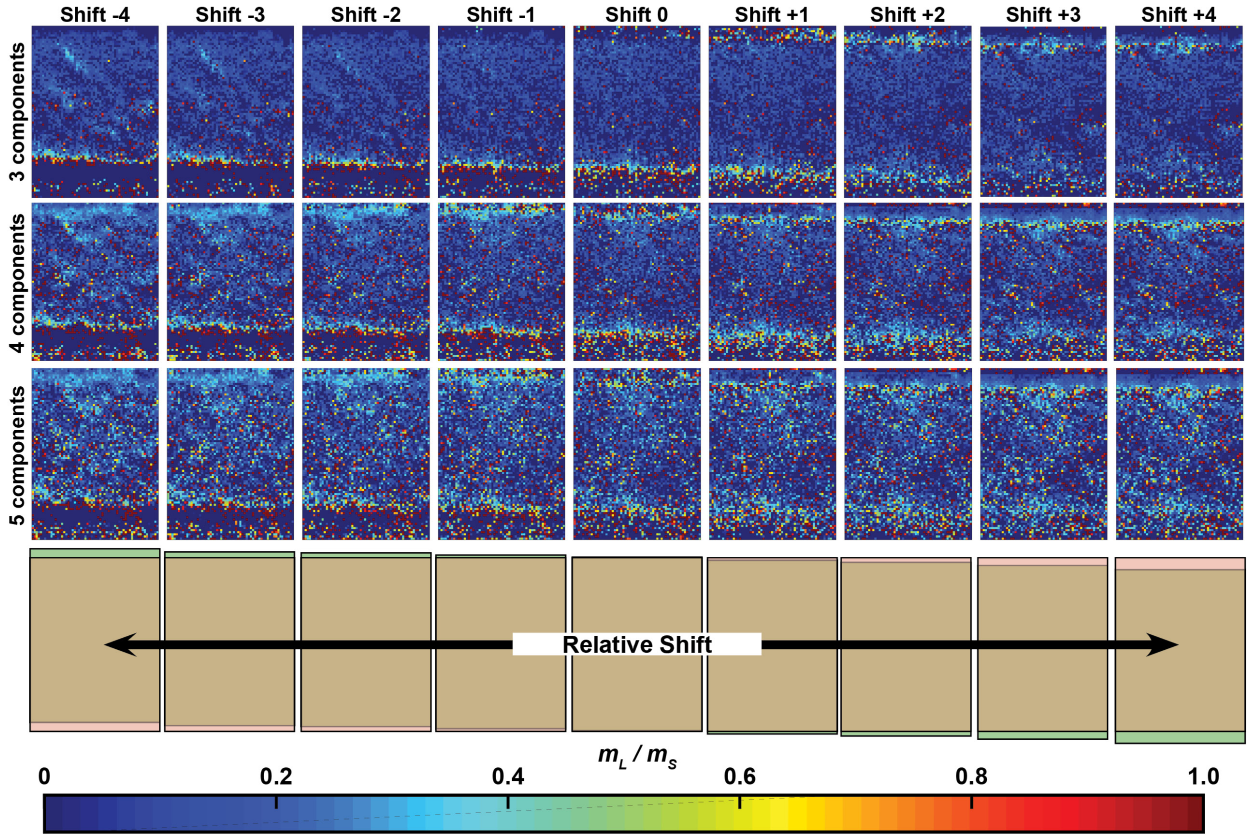

FIG. 16. $m_L/\tilde{m}_S$ real-space maps for three, four, and five component reconstructions subjected to an artificial spatial shift between the chiral plus and chiral minus scans. The shift direction was perpendicular to the interface, as depicted below.

features tend to disappear at a shift of 0, implying that the drift correction routines were accurate to within one pixel. We further observe that, despite even very large shifts, the observed enhancement of $m_L/\tilde{m}_S$ at the interface remains largely intact. We interpret this to arise from the relatively large spread of the white line ratio change over the interfacial region, due to the structurally rough nature of the interface as discussed in the manuscript. Thus, we feel quite confident that large spatial drifts in excess of one pixel can be excluded as a cause for the $m_L/\tilde{m}_S$ interfacial enhancement, and that sub-pixel drifts are insufficiently strong to account for it on their own.

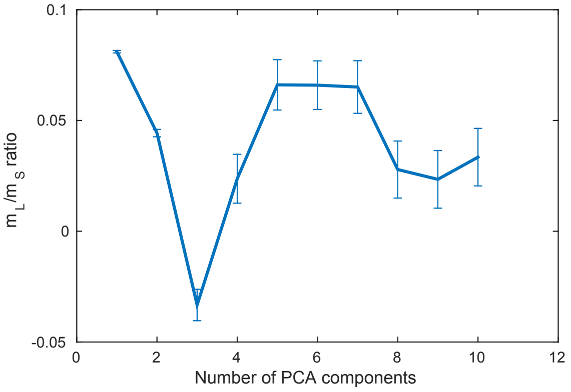

FIG. 17. The average bulk m_L/m_S and its standard error estimated as a function of the number of principal components.

STATISTICAL SIGNIFICANCE OF THE m_L/m_S ENHANCEMENT AT THE INTERFACE

As scree plots, Fig. 12, have suggested, the variance of the components after the fourth one is very low. Furthermore, Figs. 13 and 14 show that in both datasets the first seven principal components have very similar shapes, while the lower-variance components starting with the eighth and onwards differ significantly between the two datasets. This suggests that these components are not likely to be representative of a significant physical component in the datasets, rather that they represent a variance originating from the noise that is constrained to be orthogonal to the higher components. That would suggest that the number of significant components in the datasets should be in the range between 4 to 7. Below this range, the data risk being too compressed while above it they merely add noise. This qualitative expectation is well supported by a plot of an average $m_L/\tilde{m}_S$ ratio from the bulk region (columns 5 to 60) as a function of the number of components, shown in figure 17. The extracted $m_L/\tilde{m}_S$ stays practically constant within the statistical error bar when extracted from datasets reconstructed using from 5 up to 7 principal components.

Considering that the fourth component is shown to be important according to the scree plots, we have focused on the range from 4 to 7 principal components. Figure 18 presents a column-by-column analysis of the $m_L/\tilde{m}_S$ for the reconstructed datasets. In this analysis we have ignored extreme outliers, that is, pixels where the $m_L/\tilde{m}_S$ value was outside the range $(-3, 3)$. Modifying this range to $(-10, 10)$ or $(-2, 2)$ does not qualitatively influence

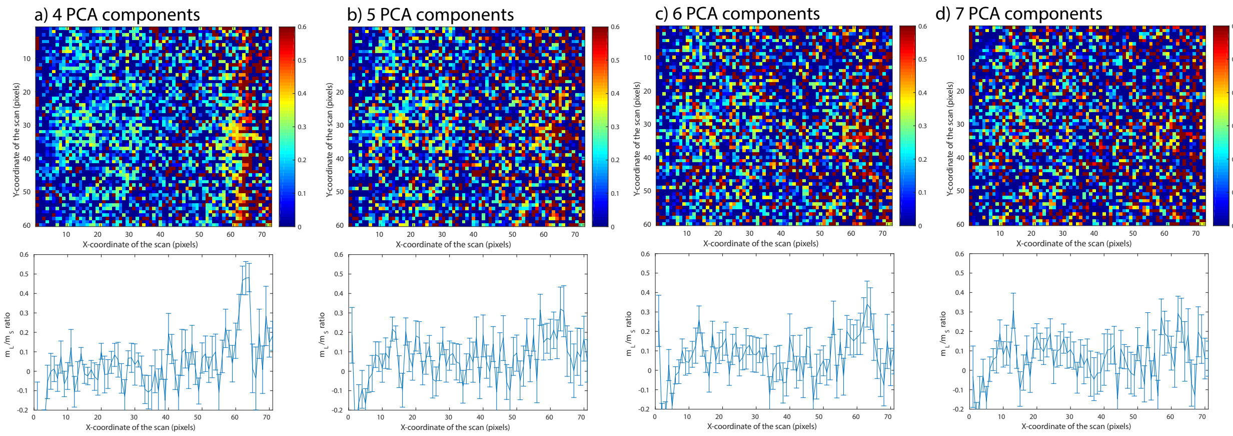

FIG. 18. Column-by-column evaluation of the $m_L/\tilde{m}_S$ ratio and its standard error evaluated from the datasets reconstructed by 4 to 7 principal components. Note the enhancement of the $m_L/\tilde{m}_S$ ratio consistently appearing as a ramp at the interface region (approximately columns 72-78).

the conclusions. For all the maps we observe a ramp-like increase of the $m_L/\tilde{m}_S$ ratio at the interface.

Figure 19 plots the average $m_L/\tilde{m}_S$ ratio from the bulk region (columns 5 to 60, the same as in Fig. 17) and from the interface region (here represented by columns 62 to 65). It can be seen that the interfacial enhancement of the $m_L/\tilde{m}_S$ ratio is well above the error bars given by standard error for up to 7 principal components. As discussed above, we have sufficient confidence that, starting from the seventh principal component, we are in a region where we can claim that every additional component adds little if any physical information and primarily enhances the noise.

STRUCTURE MODEL OF THE INTERFACE

MgO/Fe structure was prepared as presented in Fig. 20. Along c-axis two unit cells of MgO are combined with four unit cells of bcc Fe. Fe ab-plane is rotated by 45 degrees with respect to MgO basal plane. At an interface Fe atoms are placed directly over O atoms. The eventual diffusion of O into Fe region is not considered. Unit cell repeats periodically in all three dimensions. It generates the model of MgO/Fe bilayers repeating themselves along c-axis and infinite in ab-plane. Structure is fully relaxed, it means that volume, c/a

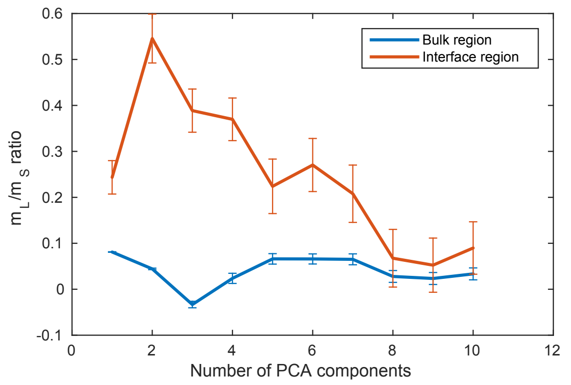

FIG. 19. Average m_L/m_S ratios their standard error bars from bulk (columns 20-70) and interface region (columns 72-78) evaluated as a function of the number of principal components used in reconstruction of the datasets.

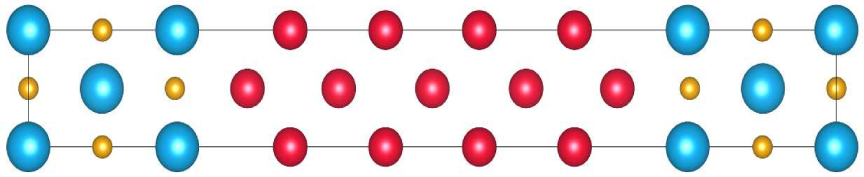

FIG. 20. The side view of considered MgO/(Fe bcc) model, side view in ac-plane. The largest blue spheres represent Mg, the smallest yellow ones O, and the medium red ones Fe.

ratio and Wyckoff positions are optimized. The highest P4/mmm symmetry is found with 11 inequivalent atomic positions, see Tab. I. Optimized lattice parameters are a = 2.969 Å and c = 23.576 Å. The VESTA code [8] is used for visualization of the crystal structure.

The full-potential linearized augmented plane-wave method (FP-LAPW) as implemented in the WIEN2k code [9] is used for both the initial optimization of the structure and following calculations of magnetic properties. Muffin-tin radii are 1.97 a.u. for Mg (1 a.u. = 0.529178 Å), 1.87 a.u. for O and 2.11 a.u. Fe. The Perdew, Burke, Ernzerhof form (PBE) [10] of the exchange-correlation potential is selected. Plane wave cut-off parameter RK_{max} is set to 7. Relativistic effects are included with the second variational treatment of spin-orbit coupling. The total energy convergence criterion is set to 10^{-8} Ry. In the irreducible wedge of the Brillouin zone the 630 k-points are used ($39 \times 39 \times 5$ mesh).

TABLE I. Atomic coordinates of considered MgO/(Fe bcc) structure with P4/mmm symmetry (sg. 123).

Atom	x y z	Atom	x y z
Mg	0.0 0.0 0.0000	Fe	0.5 0.5 0.2711
O	0.5 0.5 0.0000	Fe	0.0 0.0 0.3241
Mg	0.5 0.5 0.0909	Fe	0.5 0.5 0.3840
O	0.0 0.0 0.0915	Fe	0.0 0.0 0.4420
Mg	0.0 0.0 0.1841	Fe	0.5 0.5 0.5000
O	0.5 0.5 0.1813		

PROJECTED DENSITIES OF STATES

Figure 21 summarizes the electronic structure calculations and the local variations of the electronic structure nearby the interface. It can be seen that the projected density of states (DOS) of oxygen atoms differs depending on the closeness of the O atoms to the interface. Only p-DOS is plotted, since this one is mainly responsible for the signal seen in the oxygen K-edge. The third layer of oxygens has a DOS very close to the bulk MgO. The first layer, closest to the interface is rather different and the second layer shows features in between of the two, but closer to the bulk-like DOS. Therefore it appears that the modification of local electronic structure of oxygens due to interface is localized to the two closest atomic layers. Nevertheless, these two atomic layers provide a likely explanation for the appearance of residual oxygen K-edge signal in the fit of the spectra reported in Fig. 10.

Qualitatively similar picture is also observed for the projected DOS of iron atoms. The layer closest to the interface has strongly modified electronic structure. This modification is much reduced in the second to fifth layer, except for the features about 2-4 eV under the Fermi level. The fifth Fe layer has a DOS indistinguishable from bulk iron. This provides an explanation for the strongly localized change of the magnetic properties of the individual Fe layers reported in the main text.

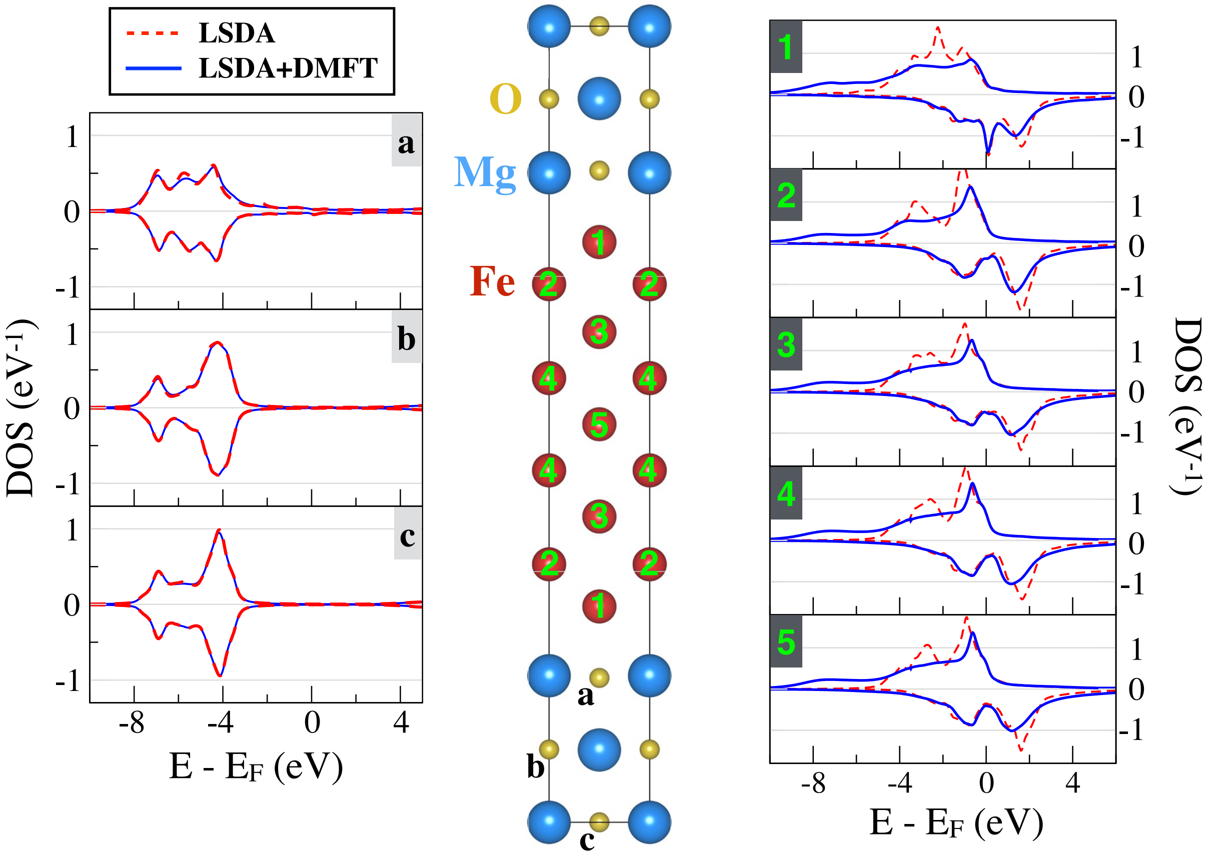

FIG. 21. (left) Projected DOS of oxygen p-states as a function of the atomic layer. Results originate from density functional theory (DFT) without considering dynamical correlations. (right) Projected DOS of Fe atoms as a function of layer, evaluated by DFT calculations (red dashed lines) and DMFT calculations (blue full lines).

ESTIMATION OF SPATIAL RESOLUTION IN THE EXPERIMENT

We estimate computationally the spatial resolution of the STEM-EMCD reported in the main text. The initial probe broadens as it propagates and inside the crystal it also scatters, which can broaden it further.

An initial FWHM probe size for a 300 keV beam with convergence semi-angle α = 2.5 mrad is estimated to be 0.53 $\lambda/\alpha \approx$ 4.5 Å. Total probe size is approximately 8 Å due to finite source size broadening. However, the finite source size broadening can be approximately modeled as an incoherent sum of diffraction-limited probes distributed around the optical axis of the microscope with a width of the distribution representing the source size

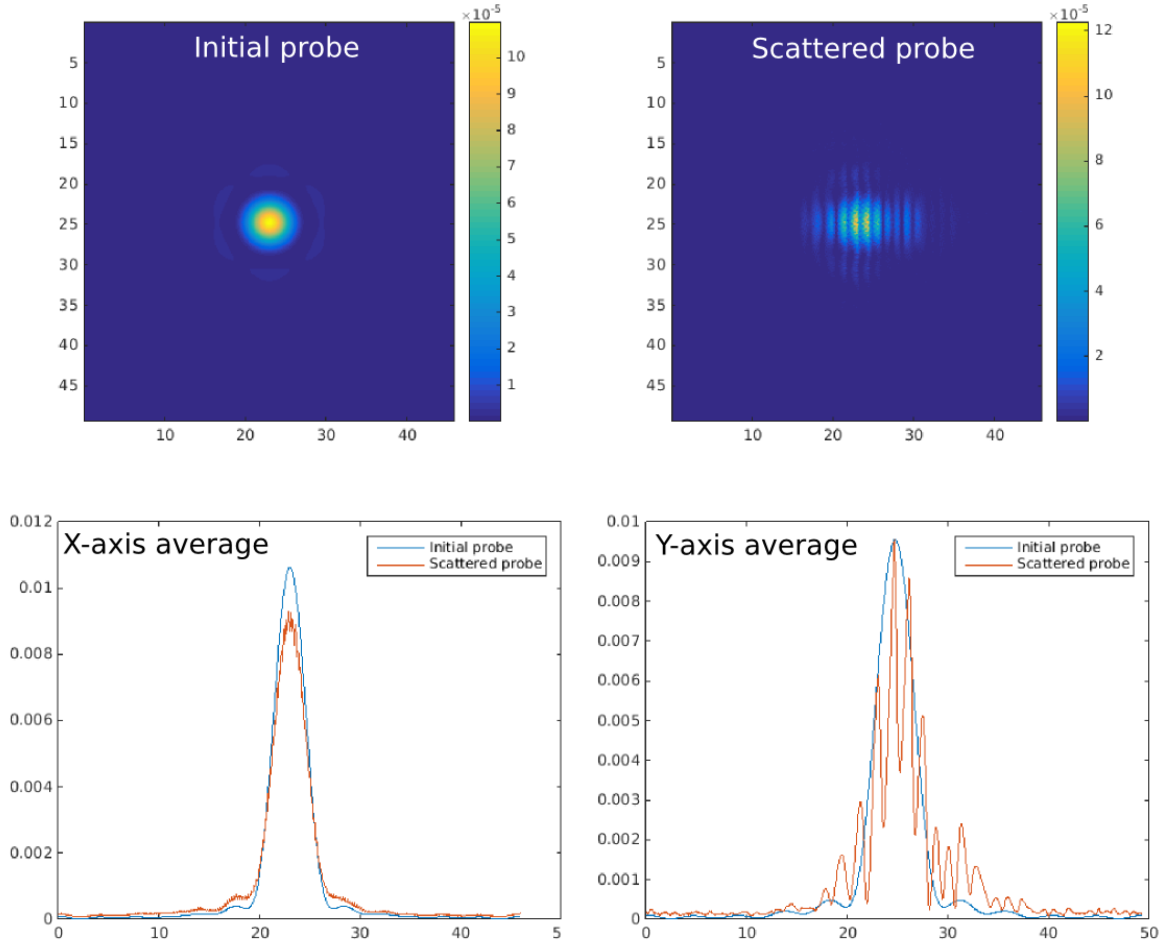

FIG. 22. Multislice simulation of the beam broadening before and after passing through 100 nm of bcc-Fe oriented in a 2-beam orientation.

broadening. This incoherence allows us to obtain an estimate of the probe broadening due to diffraction effects just by passing a single diffraction-limited probe through the crystal.

We have done simulations of the electron probe with a multislice method. The structure model was a large 148-atom orthogonal supercell of bcc-Fe with its c-axis oriented parallel to a zone axis $(0, 7, 5)$, which corresponds to a direction tilted approximately 10° from the $(0, 1, 1)$ zone axis, representing a systematic-row conditions very close to the experimental ones. The incoming beam, with acceleration voltage and convergence angle set as in the experiment, was tilted to a two-beam condition with $\mathbf{G} = (2, 0, 0)$ by about 6.8 mrad in the x-direction. Figure 22 compares the initial probe (top left) and scattered probe (top right) and the averages of their intensities along the x, y-directions. The channeling of the

probe along the lattice planes is well visible as a periodic modulation of the probe along the x-direction. On the other hand, in the y-direction the probe shape is barely modified by the scattering. Overall, despite that the probe develops heavier tails due to the scattering, its FWHM remains practically the same even after passing through 100 nm of the crystal.

Although this finding might appear counter-intuitive, it is easy to explain qualitatively. The depth of focus of a probe scales with inverse square of the convergence angle and it is approximately given by a formula 1.77 λ/α^2, which for 300 keV beam and 2.5 mrad convergence semi-angle gives approximately 560 nm. Hence, it is expected that a probe with such small convergence angle stays in focus. Moreover, our simulations show that the scattering does not significantly broaden the probe. The two-beam orientation of the crystal manifests itself in the probe only as a periodic intensity modification with periodicity of the lattice planes (channeling effect) and via an appearance of an asymmetric shoulder.

We conclude that the FWHM of the probe is not likely to broaden significantly beyond its initial FWHM. Therefore the spatial resolution of the STEM-EMCD experiment should remain close to 8 Å, safely below one nanometer.

* thomas.thersleff@mmk.su.se; Present address: Stockholm University, Department of Materials and Environmental Chemistry (MMK), 10691 Stockholm, Sweden.

[1] T. Thersleff, J. Rusz, S. Rubino, B. Hjörvarsson, Y. Ito, N. J. Zaluzec, and K. Leifer, Sci. Rep. **5**, 13012 (2015).

[2] J. Gazquez, M. Varela, D. Petti, M. Cantoni, C. Rinaldi, S. Brivio, and R. Bertacco, J Mater Sci **46**, 4157 (2011).

[3] V. Serin, S. Andrieu, R. Serra, F. Bonell, C. Tiusan, L. Calmels, M. Varela, S. J. Pennycook, E. Snoeck, M. Walls, and C. Colliex, Phys. Rev. B **79**, 144413 (2009).

[4] C. Wang, A. Kohn, S. G. Wang, L. Y. Chang, S.-Y. Choi, A. I. Kirkland, A. K. Petford-Long, and R. C. C. Ward, Phys. Rev. B **82**, 024428 (2010).

[5] C. Colliex, T. Manoubi, and C. Ortiz, Physical Review B **44**, 11402 (1991).

[6] R. F. Egerton, *Electron energy-loss spectroscopy in the electron microscope* (Springer, 2011).

[7] M. Hubert, P. J. Rousseeuw, and K. Vanden Branden, Technometrics **47**, 64 (2005).

[8] K. Momma and F. Izumi, Journal of Applied Crystallography **41**, 653 (2008).

[9] P. Blaha, K. Schwarz, G. Madsen, D. Kvasnicka, and J. Luitz, "WIEN2k, An Augmented Plane Wave + Local Orbitals Program for Calculating Crystal Properties," (2001).

[10] J. P. Perdew, K. Burke, and M. Ernzerhof, Phys. Rev. Lett. **77**, 3865 (1996).
